# Ideological self-selection in online news exposure: Evidence from Europe and the US

**DOI:** 10.1126/sciadv.adg9287

**Published:** 2024-09-13

**Authors:** Frank Mangold, David Schoch, Sebastian Stier

**Affiliations:** ^1^ Department Computational Social Science, GESIS – Leibniz Institute for the Social Sciences, Unter Sachsenhausen 6-8, 50667 Cologne, Germany.; ^2^ School of Social Sciences, University of Mannheim, Mannheim, Germany.

## Abstract

Today’s high-choice digital media environments allow citizens to completely refrain from online news exposure and, if they do use news, to select sources that align with their ideological preferences. Yet due to measurement problems and cross-country differences, recent research has been inconclusive regarding the prevalence of ideological self-selection into like-minded online news. We introduce a multi-method design combining the web-browsing histories and survey responses of more than 7000 participants from six major democracies with supervised text classification to separate political from nonpolitical news exposure. We find that political online news exposure is both substantially less prevalent and subject to stronger ideological self-selection than nonpolitical online news exposure, especially in the United States. By highlighting the peculiar role of political news content, the results improve the understanding of online news exposure and the role of digital media in democracy.

## Teaser

Exposure to political news is less prevalent and shaped more by ideological self-selection than nonpolitical online news.

## INTRODUCTION

Among the various threats that liberal democracies are facing around the globe, the perhaps most foundational question of contemporary political communication is: Does the ever-expanding supply of online news sources provide the kind of shared space for political information that is necessary for well-functioning democratic societies? Or do digital media provide a fertile ground for so-called “echo chambers” in which ideologically like-minded citizens isolate themselves? For one, these questions concern normative ideals about the nature of information flows in the digital age (*1*, *2*). For the other, they are hard to tackle without distinguishing online users’ exposure to political news content from their exposure to nonpolitical news (sports, weather, traffic, etc.). Isolating politically relevant news exposure enables more meaningful empirical analyses of liberals’ and conservatives’ ideological self-selection into politically congruent news content (*3*–*5*). This study innovates by using a combination of web-browsing histories with surveys and supervised machine learning to disentangle political from nonpolitical online news exposure and how both are characterized by ideological selectivity in different ways, including the question of how ideological selectivity differs across Europe and the US.

### Digital high-choice media environments: A threat to democracy?

The proliferation of information sources due to the advent of online media and intermediaries like social networking sites has been described by scholars as a “high-choice media environment” (*6*). Despite profound cross-national differences in the supply of news and the extent to which media environments are digitized (*7*, *8*), research from various parts of the world has observed the same core mechanism: “The greater the media choice, the more selective people have to be, and the more selective people have to be, the more important their preferences become” (*6*). Such digital high-choice media environments might affect news use in two major ways. First, there are fears that citizens would get exposed less to news and public affairs information, given widespread preferences for entertainment and a growing disdain of politics. Amid an abundance of media choices allowing citizens to opt out of news, an important pillar of democracies, an informed citizenry, might get eroded (*9*). Likewise, studies have shown that news use is increasingly concentrated among politically interested citizens (*6*). Second, ideological selectivity might increase because the proliferation of partisan online media has provided citizens with unprecedented opportunities to predominantly consume news content that aligns with their political preferences (*10*). An ideological sorting of news audiences—reminiscent of so-called echo chambers, an information environment in which primarily like-minded content is consumed at the expense of non-congruent perspectives (*11*)— threatens the common ground of democracy. If citizens only use news that reinforce their ideological views and, in particular, one-sided coverage of public affairs, then they may end up inhabiting alternative political realities. Negative downstream effects like a proliferation of misinformation, an erosion of the legitimacy of policy decisions due to a lack of cross-cutting perspectives, or affective polarization may arise (*12*).

A comprehensive understanding of modern news environments requires considering nonuse of news and ideological selectivity in conjunction. Both are important concerns in light of normative theories of an informed democratic public sphere (*13*) and have gone hand in hand with a more general skepticism of social media and other online intermediaries (*1*, *2*). Their algorithmic recommender systems have often been suspected of biasing media diets in line with citizens’ preferences (*14*, *15*), although recent empirical evidence has indicated that intermediaries actually tend to increase the amount and diversity of news exposure (*16*–*19*). Another important concern in scholarly debate is the question of how political ideology interacts with the arguably most important covariate of news exposure: political interest. While there is a broad consensus that political interest stimulates news use, some scholars have argued that politically interested citizens are ideologically less selective news users (*20*), whereas others see reason for the opposite pattern (*13*). Last, the digitization of media systems requires reconceptualizing established typologies for cross-country analysis. While “political parallelism” of news outlets and ideological selectivity on behalf of citizens have traditionally been located in Southern European media systems like Spain or Italy rather than in the US (*21*, *22*), the differences have been reduced in recent decades. The US has shifted toward an unprecedented “polarized liberal” model (*23*, *24*), implying higher ideological selectivity than in Europe (*7*, *25*). Yet due to the limited accuracy of self-reported media use (*26*, *27*) and a narrow focus of web browsing-based studies on partisan media use in isolated countries, most often the US (*4*, *28*, *29*), there is still no robust empirical investigation of ideological selectivity in digital high-choice media environments to date.

### Methodological challenges in studying news exposure

Digitization has not only fundamentally changed citizens’ news use, it also creates previously unknown opportunities for measuring media-related behaviors. Although still prevailing, survey measures have always suffered from the drawback that people notoriously overreport news use due to its social desirability (*30*). The limited validity and reliability of self-report measures is aggravated in the online sphere. The relative ease by which people can switch between the various content options online makes it particularly difficult to accurately recall media use (*26*, *27*), not least when people arrive at news through intermediaries like social media or search engines (*31*, *32*). Another deficit of surveys is their limited coverage. Even the state-of-the-art list-frequency survey approach (*33*) makes it impossible to capture more than a handful of news outlets. Therefore, researchers miss out on partisan news use in the long tail of online niche media (*34*).

To mitigate the deficits of self-report measures of media exposure, a growing body of research relies on passively collected data to directly observe participants’ online behaviors in natural real-world settings (*35*). Any observation of online behaviors naturally only measures parts of citizens’ overall media use. Yet, at a time when growing proportions of media use have been shuffled from the offline to the online sphere, online media already predominate among certain parts of the population. Likewise, many fears about polarization in liberal democracies have revolved around the online component of contemporary media environments (*29*, *36*). Nonetheless, because research using behavioral data has found only limited ideological segregation of news audiences online, a consensus has emerged that citizens with different ideological leanings mostly continue to share news exposure with each other. A picture is emerging that the modal online user obtains news and political information from a diversity of sources and that partisan media occupy only a small fraction of her news diet (*3*, *5*, *17*). The strongest advocates against selective exposure theory argue that fears about ideological selectivity in online news exposure are not just exaggerated but essentially a myth, even in the US (*37*). However, for two reasons, previous studies have underestimated the role of ideological selectivity in online media environments.

First, the most important reason for citizens to engage in partisan selective exposure while avoiding ideologically disagreeable information is to reduce cognitive dissonance and protect their political convictions (*25*, *38*, *39*). Nonetheless, predominant operationalizations in the field rarely tested this theoretical premise directly on political content but relied on aggregated domain-level information. Therefore, most research did not distinguish political from nonpolitical news use [exceptions are (*4*, *29*)]. Simply put, news providers offer coverage on various topics like entertainment, sports, or weather, for which users have little reason to engage in ideological selectivity (*40*), although some specifically engaged partisans may naturally extend their selectivity more to nonpolitical domains of life than other citizens do (*4*, *13*).

Second, in line with traditional models of television use, understanding selective exposure online requires conceptualizing news selection as a two-stage process (*41*–*44*). User preferences do not inevitably come into play at the stage of initial news source selection. Instead, users may often encounter a diversity of content options while surfing the web, yet still eventually decide to only spend longer periods of time on and engage with ideologically more agreeable news content. This mechanism has been obscured because even behavioral studies have used artificially low temporal thresholds for counting news website visits as news use episodes [an exception is (*43*)]. For instance, commercial audience data by companies like Comscore or Nielsen counts someone as having used a news website if they access it for just 3 seconds (s) by default (*5*, *37*). Consequently, the data also contain many false positives (from a substantive rather than a statistical viewpoint), as extremely short news visits preclude any form of real engagement with the content (*18*, *34*) and any gains in knowledge and participation (*39*, *42*, *45*, *46*).

To investigate ideological self-selection in online news exposure across countries, we use the most comprehensive collection of individual-level web-browsing data thus far. Our multi-method design combines the browsing histories of more than 7000 participants from six major democracies with the complementary advantages of surveys (*35*). To classify news content, we scraped the online news articles seen by participants and used supervised text classification to distinguish political from nonpolitical news exposure. The data were collected in France, Germany, Italy, Spain, the UK, and the US from March to June 2019. The country-comparative design goes beyond previous studies that have been confined to isolated cases, most often the US, instead of transatlantic comparisons. The data cover different types of media and political systems as well as different civic cultures (for details, see table S2). Thus, if results are consistent across our country sample, then they likely apply to developed democracies in general (*47*). While the linkage with surveys is essential for taking into account online users’ ideological leanings and relevant individual-level factors like political interest, these linked data come with the trade off that the underlying recruitment process is not probability based. Accordingly, our samples deviate on some characteristics from the general population (see table S1, also the validations against external benchmarks in section S5). While web tracking is the only way to capture online content exposure, data at the granular level needed for our research questions are only available through browser plugins on desktop computers and laptops. Therefore, our analysis misses out on news exposure via smartphones and associated apps, besides traditional forms of news use, e.g., via television or newspapers.

## RESULTS

News accounted for only a small proportion of total website visits in all countries, with baseline probabilities of a news visit ranging from 0.8% (in the US) to 2.7% (in Spain; for details, see section S9.1), in line with other recent studies using web-tracking data (*18*, *48*, *49*). More than 85% of participants per country visited a news website at least once during the 3-month period covered by our study. In the following, political news is defined and measured with a text classification model as content that is related to either polity (e.g., political institutions, and democracy), politics (e.g., elections, political actors, and scandals), or policy (e.g., regulation or legislation with regard to substantive issues, which excludes non-policy aspects like crime reports). We thereby follow comprehensive definitions from political science (*50*) and address the criticism that previous US studies have operated with overly lenient definitions of political news (*4*).

### Prevalence of political and nonpolitical online news exposure

**Fig. 1.**
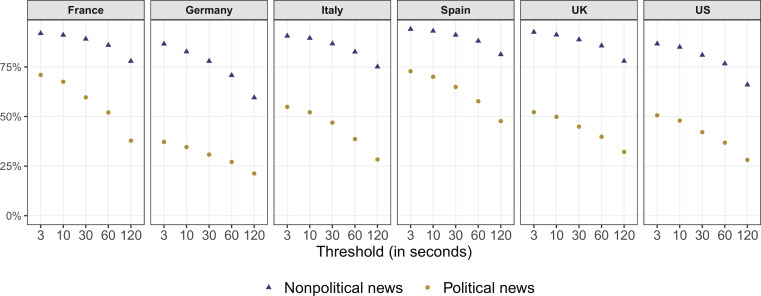
News website visitors with exposure to political and nonpolitical news. Share of participants who had at least one visit to either a political or nonpolitical news article.

Figure 1 breaks down the online news audience within each country dataset in terms of the shares of news website visitors with exposure to political and nonpolitical news articles, contingent on the range of temporal thresholds that have previously been considered for counting news visits as news use episodes. The 3-s threshold is the default website visit duration in studies relying on audience data by companies like Comscore. The 120-s threshold can be regarded as a conservative estimate of what constitutes a meaningful news episode (*43*), while we also apply intermediate thresholds of 10, 30, and 60 s.

The results in Fig. 1 show that exposure to political online news was overall less prevalent than exposure to nonpolitical online news from the outset. While the vast majority of participants got exposed to nonpolitical news, the share of participants with exposure to political news was lower by 20 percentage points or more in all countries, even with the liberal 3-s threshold. The longer thresholds naturally decreased the share of the sample with at least one political or nonpolitical online news website visit. Still, the share of nonpolitical news visitors generally remained above 60%. In contrast, 50% or more of the participants in each country did not have a single visit of a political news article longer than 120 s. Overall, the results demonstrate that, compared to nonpolitical online news, exposure to political online news is substantially less widespread across the sample.

### Prevalence of ideological self-selection online

We follow “audience-based” research in political communication that determines the editorial ideological alignment of a given online news outlet based on the political leanings of its users (*51*). As our central metric of an outlet’s editorial slant, we calculated the mean ideology of its users, weighted by their number of visits to the outlet. Various operationalizations of these news outlet alignment scores were applied in web-tracking–based research on ideological self-selection in the US (*4*, *28*, *29*).

**Fig. 2.**
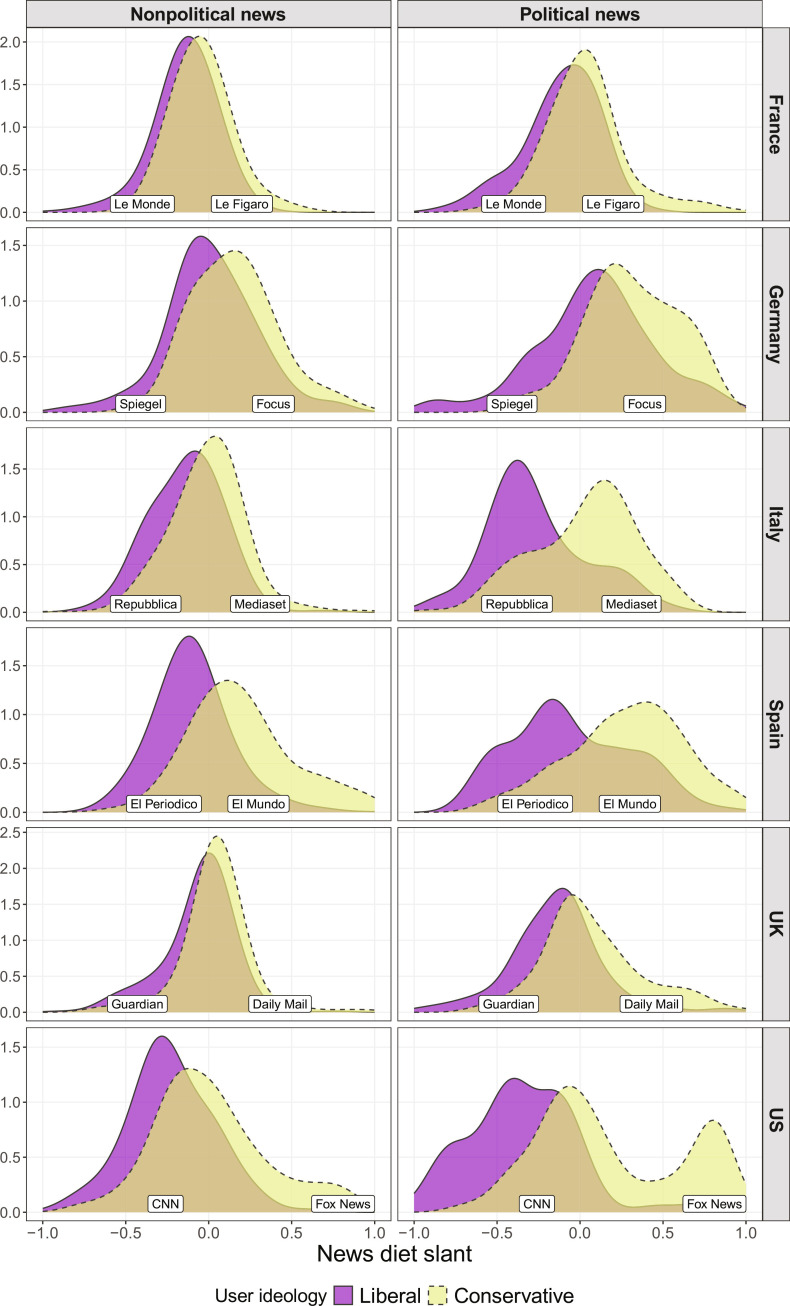
Distributions of online news diet slant of liberals and conservatives. Density distributions obtained with the 120-s visit threshold. Using the 3-s threshold produced similar but less pronounced differences between countries and across political and nonpolitical news (fig. S12). Online news diet slant is the mean ideological alignment of the news websites visited by each user; outlet alignment scores are audience-based estimates of editorial slant (details in Materials and Methods). Alignment scores of the top 15 news sites (in % reach) in each country are shown in fig. S2.

To demonstrate how news outlets are ideologically aligned in different media systems, Fig. 2 plots the distribution of the news diet slant of the individual exposure of study participants with a self-reported liberal or conservative ideology. The rows represent the countries, and the columns differentiate political and nonpolitical online news exposure. As a visual reference point, the most widely visited left-leaning and right-leaning news outlets in each of the six countries are included. News diet slant scores close to −1 and 1 represent ideologically one-sided news exposure in the sense that those participants’ online news diets are dominated by visits to news websites with a strong left- or right-leaning ideological alignment, respectively.

The distributions in Fig. 2 reveal three main patterns: (A) Compared to nonpolitical news, the news diet slant distributions for political news were more widely dispersed in all countries. Liberals and conservatives were therefore less likely to read the same online news articles when these were about political topics. (B) Among the European countries, the ideological slant of liberals’ and conservatives’ political online news exposure diverged most strongly in Spain and Italy, in line with their traditional classification as polarized media systems (*22*). (C) The US stands out due to a unique asymmetry of US liberals’ and conservatives’ political online news diet slant. There was a pronounced concentration of US conservatives’ political online news exposure at the right end of the ideological spectrum. Their political online news diets most closely resemble the notion of echo chambers, as many conservative study participants were heavy users of outlets like Fox News or fringe outlets further right while being detached from the ideological center of the US media system.

We next bring in the time thresholds to assess whether the intensity of news episodes matter in the extent of ideological self-selection in online news. We formally calculate three different metrics from previous literature to assess whether existing measures of ideological self-selection produce consistent results: (A) isolation, which is the most common measure of ideological segregation and captures the extent to which liberals and conservatives do not share exposure to news websites with each other (*5*, *17*, *52*); (B) Simpson’s *D*, which is a more sophisticated measure of news diet diversity that captures how equally online users distribute their visits across more liberal, centrist, and conservative news outlets (*16*, *19*, *53*); and (C) the standard deviation (SD) of online news diet slant (*29*). The online news diet slant measure captures the overall ideological leaning of each participant’s news website visits. Accordingly, its SD quantifies the extent to which users have an either strong left- or right-leaning alignment, as opposed to visits of centrist news outlets or ideologically mixed (“cross-cutting”) visits (*29*). All three descriptive statistics are scaled similarly from of 0 to 1.

**Fig. 3.**
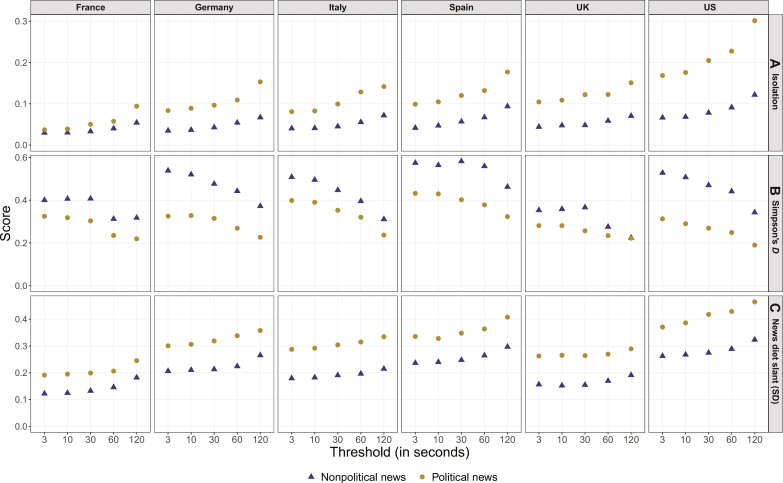
Descriptive statistics of ideological self-selection. The rationale of the statistics is explained in the section "Prevalence of ideological self-selection online." Simpson’s *D* scores are average scores across users. Details and formulas in Materials and Methods.

The results in Fig. 3, row A, demonstrate that, in line with previous behavioral studies of ideological self-selection, the isolation scores for nonpolitical online news obtained with the 3-s threshold were low to very low in all countries. The isolation scores for political online news were generally higher, especially with the 120-s threshold. While consistent across countries, the pattern was most pronounced in the US, where the isolation score for political online news gradually approached the value of 0.3 that has traditionally been regarded as an indication for the existence of enclaves in research of segregation (*52*).

Rows B and C show that the higher isolation scores corresponded with a lower ideological diversity and greater role of partisan media in participants’ political online news diets, especially with the longer temporal thresholds and in the US. The sole exception in terms of news diet diversity was the UK, where many participants concentrated their nonpolitical online news exposure on the BBC. More generally, the Simpson’s *D* scores for political news indicate that low numerical isolation scores should not be conflated with widespread cross-cutting online news exposure. The scores range from about 0.4 (for Spain with the 3-s threshold) to less than 0.2 (for the US with the 120-s threshold), meaning that a single ideological camp (left, center, and right) generally occupied more than 75% and up to more than 90% of the average political online news diet. Still, the SD of online news diet slant generally remained below 0.5, even in the US and with the 120-s threshold. Therefore, participants did not exclusively obtain political news from the most radical partisan media, but more ideologically modest outlets continued to play a substantial role among their political online news diets. Together, the three measures draw a consistent portrait of higher ideological self-selection in case of political news and longer news episodes.

### Individual-level analysis of ideological self-selection

**Fig. 4.**
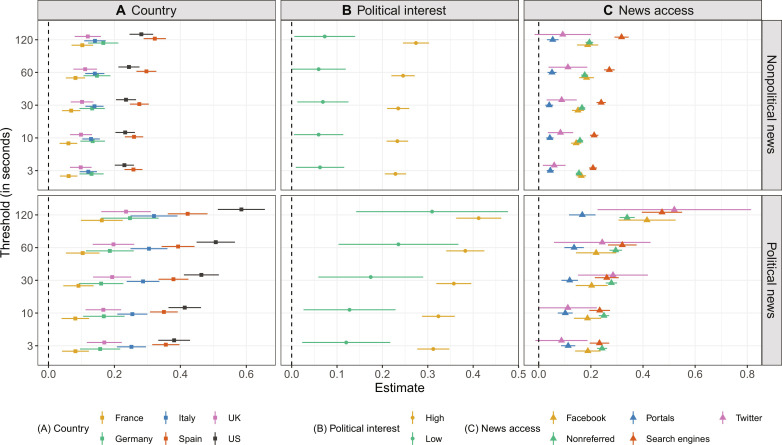
Conditional differences in liberals’ and conservatives’ online news diet slant. Regression coefficients and 95% confidence intervals from linear multilevel models obtained by treating participant ideology as a categorical predictor (details in Materials and Methods). Demographics (gender, age, and education) as well as political extremity and total number of website visits are included as controls but not reported. Full results and additional significance tests for the conditional differences can be found in figs. S7 and S8.

For an integrated analysis of how online news diet slant varies not only by countries but also by citizens’ political interest and news access modes, we use multilevel regression analysis. The aim is to rule out that differences in the news exposure of liberals and conservatives merely reflect that they are populations with different demographic characteristics (e.g., older citizens tend to be more conservative) (*16*, *29*, *43*, *54*). It specifically deals with the nested nature of our data by considering that individual news visits (i.e., level 1 units of analysis) are nested within participants (i.e., level 2 units), which are, in turn, nested within countries (i.e., level 3 units) (*27*, *49*, *53*, *55*). Figure 4 summarizes the multilevel regression’s main results. The regression coefficients (displayed on the *x* axis) capture the difference between the mean ideological slant of liberals’ and conservatives’ online news diets, after adjusting for the control variables. The two rows represent the differences between political and nonpolitical online news exposure for different temporal thresholds on the *y* axis. The three columns depict the theoretically relevant differences across countries, levels of political interest, and news access modes.

Figure 4 shows four main results. First, especially with the longer thresholds, the coefficients in the bottom half of Fig. 4 were consistently higher than their counterparts in the top half. In line with the descriptive findings, there is robust evidence that political news visits and, in particular, longer political news visits are shaped more by ideological selectivity than nonpolitical news visits. Second, the country-specific coefficients in the bottom left panel were substantially more varied than the coefficients in the top left panel, as ideological selectivity in political online news exposure differed more strongly between media systems compared to nonpolitical online news exposure. Again, the US-specific coefficients for political online news showed the strongest difference between the slant of liberals and conservatives’ online news diets, followed by Spain and Italy.

Third, politically more interested participants were overall more ideologically selective than those with low political interest, especially when it comes to political news articles. Furthermore, politically interested participants specifically stood out for the shorter temporal thresholds, meaning that their political online news diets were dominated by selective exposure to ideologically like-minded sources to begin with. Last, the results for news access modes show that the ideological slant of liberals’ and conservatives’ political online news exposure differed the least when news was accessed via portals and most strongly when accessed via search engines or when not reached via intermediaries, i.e., news websites were directly accessed without a referral. Political news access via Facebook and Twitter gravitated between these poles, with increasing ideological selectivity for those social media referrals that resulted in longer political news visits.

## DISCUSSION

Amid vivid public and scholarly debate about the democratic impact of digitization, our study enables a better understanding of how ideological selectivity plays out in online media environments. Theoretically, we argue that nonuse of political news and ideological selective exposure cannot be considered in isolation from each other. Going beyond research that has considered single cases in isolation, most notably the US, we combined web-browsing histories with surveys of the same participants and supervised text classification to identify political online news.

Our first main finding is that political online news exposure was substantially less prevalent than nonpolitical online news exposure. Hence, a substantial share of online news exposure is of restricted democratic value in the first place. More generally, the results pointed to a divide that is often overlooked: The vast gap between citizens for whom politics is an important part of their lives and those who are politically apathetic (*13*, *56*). Our second main finding is that political online news exposure was also characterized by substantially higher levels of ideological self-selection, especially longer visits of political news articles. Both results highlight that political online news exposure, particularly more intensive episodes that enable an active engagement with content, needs to be theoretically and empirically separated from nonpolitical online news exposure. A generally low diversity of political online news exposure coupled with partisan self-selection in case of longer news visits results in higher levels of ideological segregation online than found in previous research.

Our third set of findings is derived from our unique cross-country comparison. On the one hand, both the lower prevalence and higher ideological self-selection into political news exposure were consistent across the US and the European countries. On the other hand, we found that echo chamber-like structures play a more prominent role in the US and, in particular, among US conservatives who got their political news predominantly from Fox News or outlets like Breitbart News even further right that have a more pronounced ideological slant (*3*, *4*, *12*, *57*). Much recent research has portrayed the US as a unique media system where the transition from low-choice to high-choice media environments has proceeded the furthest. Our results contribute to this discussion by showing that important transatlantic commonalities exist alongside various differences. Neither nonuse of online news in general nor political online news was more pronounced in the US than in the European countries. What ultimately set the US apart was higher ideological selectivity in political news exposure. Because the result clearly deviated from traditional media system classifications (*22*), it seems that the US has drifted toward a more ideologically polarized media system (*24*). Apart from that, the cross-national research design revealed that it remains important to consider the differences among European media systems, as ideological self-selection was still more pronounced in Spain and Italy than in other European democracies.

Fourth, the results highlight microlevel differences in digital behavior. Online news exposure of politically interested citizens was characterized more by ideological self-selection across political and nonpolitical online news. This especially matters as politically interested citizens account for the majority of political news visits. The results also confirmed that online intermediaries reduce the slant of citizens’ political news diets compared with direct news website visits. Still, there are differences across types of intermediaries. While news access via portals and, at least for shorter news website visits, social media corresponded to reduced ideological selectivity in political news exposure, the opposite was the case for search engines. This supported that partisans often use search engines in a more goal-oriented manner (*41*, *58*).

Last, we want to mention remaining limitations and avenues for further research. Even with web-tracking techniques, exposure within smartphone apps and intermediary platforms remain blind spots. While studies have indicated that people exhibit similar levels of ideological self-selection offline and online (*17*, *23*), future studies will have to assess differences between political and nonpolitical news exposure in traditional channels like television that still play an important role in most democracies, at least among older cohorts (*36*, *59*). Furthermore, determining the ideological slant of individual news articles remains a major methodological challenge, especially in cross-nationally comparative research. The distinction of political from nonpolitical news affects the understanding of online audiences, but politics is still inherently multidimensional, indicating that the identification of specific issues in news coverage would add further nuance to our findings. Last, besides the need for comparable data collections in European countries other than those covered herein and beyond high-income democracies, more longitudinal research should be devoted to the downstream political effects of ideological self-selection in contemporary media environments.

## MATERIALS AND METHODS

### Samples

The data collection was approved by the Oxford Internet Institute’s Departmental Research Ethics Committee (reference number SSH IREC 18 004). Our analysis relies on the web-browsing histories of 7775 study participants recruited from the participant pool of the market research company Netquest that maintains online access panels with a continuous web tracking. Participants had given their informed consent and were incentivized to install tracking tools and keep them active on their desktop computers and/or mobile devices. Our surveys of the same study participants covered the most widely studied demographic correlates of news use (age, gender, education, political interest, and political ideology). Descriptive statistics of the sample composition and all used variables are available in sections S1, S2, and S4. The tracking data comprise the browser logs from 15 March to 16 June 2019, amounting to a total of 136 million website visits, after merging subsequent visits of the same URL to account for automatically reloading browser tabs. For privacy reasons, participants could pause the tracking tool at any time. The median number of active days is 71 of a maximum of 94. Despite the non-probabilistic sampling of participants, the data enable a detailed investigation of the relative differences between political and nonpolitical news exposure. While related research using web tracking has considered single cases, most often the US, in isolation, our data enable a cross-country comparison. As a validation of the samples, we show that online and offline news exposure and privacy attitudes of study participants resemble external benchmarks (section S5). Ideological self-placement was collected using an 11-point scale survey item ranging from 1 = very left to 11 = very right. We coded all right-leaning responses as 1, left-leaning responses as −1, and center responses as 0. Recoding is necessary to establish compatibility with previous research (*5*, *17*) and the calculation of descriptive statistics like segregation scores. Moreover, this approach provides a conservative standard, ensuring that results are not contingent on cross-national differences in the preferences for more extreme response categories (*7*, *23*, *60*). We constructed an additional measure of political extremity by folding the raw ideology scores to control for gradual differences in ideological strength in the multilevel analyses.

### Measures

To identify relevant news visits in our data, the top 5000 visited domains and most used intermediary platforms in each country were hand coded, covering 89% of all website visits (see section S3 for a description of the coding and summary statistics) and resulting in a list comprising 556 news domains. Only domains that provide political news were included, using the definition of political news outlined in the main paper. Following the same definition, we crawled the texts of all visited news URLs and trained a machine learning classifier to identify visits to political news articles. We describe and evaluate the text classification in more detail in section S6. For categorizing different news access modes, we followed recent web-tracking research that has inferred news access on the level of individual news visits and, more specifically, from the domains that preceded news visits in the participants’ individual browsing histories. For instance, if Facebook was the domain a participant immediately visited before visiting a news URL, news access was coded “Facebook” (*16*, *18*, *29*, *32*).

In line with previous web-tracking research (*3*, *4*, *17*, *28*), we used the audience-based method for estimating the relative ideological leaning of news outlets. This approach uses information about the composition of each news outlet’s audience as a proxy of its ideological alignment on the basis that the more left-wing (or right-wing) an outlet’s audience, the more likely it is to have a left-leaning (or right-leaning) editorial slant. Specifically, we calculate the ideological alignment score *A_ij_* for each news outlet *i* from country *j* as

$$A_{\mathrm{ij}}=I_{i}-C_{j}$$

where *I_i_* refers to the mean ideological leaning of outlet *i*’s users, weighted by their number of visits of outlet *i*. *C_j_* refers to the mean overall ideological leaning in country *j* and takes into account that some countries as a whole ideologically lean more to the left (or right) than others. It is important to highlight that the audience-based approach has advantages and drawbacks. The approach generates valid proxy measures for relative comparisons of news outlets’ ideology on an ideological continuum, is transparent, and can be replicated across countries and over time. Accordingly, the alignment scores calculated with the present data correlate highly with similar scales derived in recent US studies from Facebook or Twitter user behavior (section S9.3). Like other estimates, audience-based outlet slant scores do not provide reliable information on the absolute position of outlets on the left-right spectrum. Thus, an alignment score of 0 (i.e., the scale midpoint) should not be interpreted as indication of impartial reporting in an absolute sense or as an objective benchmark of ideological balance or lack of editorial slant [see for extended discussions, e.g., (*3*, *16*, *28*, *29*)].

### Data analysis

The “isolation” (*I*) index was originally derived in research of racial segregation (*52*, *61*). For calculating audience isolation in terms of ideology, it can be formally represented as

$$I=\sum_{i\in I}‍\frac{c_{i}}{c_{\text{total}}}\cdot \frac{c_{i}}{v_{i}}-\sum_{i\in I}‍\frac{l_{i}}{l_{\text{total}}}\cdot \frac{c_{i}}{v_{i}}$$

where $\frac{c_{i}}{v_{i}}$ is the share of conservative visits to news site *i* and $\frac{l_{i}}{l_{\text{total}}}$ and $\frac{c_{i}}{c_{\text{total}}}$ refer to the share of their total visits that liberals and conservatives devote to outlet *i*, respectively. The resulting metric is a relatively stringent conceptualization of ideological news audience polarization, capturing whether liberals and conservatives have common exposure to news websites at all, rather than simply differing in just some of their news choices (*5*, *17*). The study of Simpson’s *D* (*62*) has been specifically advanced to overcome the ambiguities of research of news diet diversity that has taken the number of visited news outlets as a proxy (i.e., bears the risk of mistaking diversity with users merely visiting, for instance, multiple conservative news sites). It can be formally represented as

$$D_{j}=1-\sum_{c\in C}‍{\left(N_{\mathrm{cj}}/N_{j}\right)}^{2}$$

where *N_cj_* represents the number of user *j*’s news visits to liberal, centrist, and conservative outlets; and *N_j_* refers to the total number of her or his news visits. To establish that lacking cross-cutting exposure is specifically reflected by low diversity scores, we followed prior audience-based research [e.g., (*16*, *49*)] by categorizing news sites with alignment scores below −0.2 as liberal, sites with scores between −0.2 and 0.2 as centrist, and sites with scores above 0.2 as conservative. We used the same categorization scheme for identifying the most widely visited left- and right-leaning news sites reported in Fig. 2. The study of online news diet slant (*S*) has been advanced in recent web-tracking research to prevent overestimating the ideological divergence of news audiences (*29*). It is defined as the mean ideological alignment of the news sites visited by each user, as weighted by the number of her or his visits to these sites

$$S_{j}=\frac{1}{N_{j}}\sum_{i\in I}‍N_{\mathrm{ij}}A_{i}$$

where *N_j_* is the total number of news visits of user *j*, *N_ij_* is the number of user *j*’s visits to outlet *i*, and *A_i_* is the ideological alignment score of outlet *i*. In practice, this means that, if a participant makes, e.g., seven visits to a news site with an alignment score of 0.75 and three visits to another news site with an alignment score of 0.90, then he or she gets an online news diet slant score of 0.795. Using the same notation, the calculations of the SD of online news diet slant can be formally summarized as

$$\text{SD}\left(S\right)={\left[\frac{1}{N-1}\sum_{j\in J}‍{\left(S_{j}-\bar{S}\right)}^{2}\right]}^{\frac{1}{2}}$$

where *S_j_* represents the ideological slant of user *j*’s online news diet, $\bar{S}$ is the average ideological slant across all users’ online news diets, and *N* is the number of users. Section S9.2 shows that the relative differences between political and nonpolitical news, temporal thresholds, and countries are similar when using the “expected squared distance” (*28*).

Last, the multilevel analysis has the key advantage over traditional single-level regressions and related tests of mean differences that it allows us to fully exploit the granularity of the cross-national web-tracking data (including the investigation of visit-level characteristics like news access modes) while simultaneously enabling accurate significance tests (across analysis levels) and establishing compatibility of the regression estimates with related research (*18*, *29*, *49*, *54*). The coefficients reported in Fig. 4 were obtained by treating participant ideology as a categorical predictor. We treated liberals as the reference group (coded “0”) and coded participants having a conservative ideological leaning “1”, such that (A) positive regression coefficients reflect that conservatives exhibit more right-leaning news diets than liberals and (B) these coefficients reflect the average difference between the slant of liberals’ and conservatives’ news diets (rather than, as the case when treating ideology as a continuous predictor, the slant difference associated with a one-unit difference in the underlying ideology scale). Further background on the data analysis is provided in sections S7 and S9.2.

### Robustness tests

We conducted several tests to probe the robustness of results against methodological choices and the compatibility of our data with previous studies. (A) The lower prevalence of political online news exposure specifically observed with the longer temporal thresholds was not due to the circumstance that participants generally spend less time on political news articles. The share of political news visits increased with the longer thresholds in all countries (see section S9.1). (B) The results were robust against the calculation of alternative descriptive statistics of ideological self-selection (see section S9.2); (C) when replacing the ideological alignment scores calculated with the present data with the ones used in recent US studies [(*3*, *29*, *49*, *51*, *63*), see section S9.3]; (D) when dropping the controls from the multilevel regressions (see section S9.4) and (E) repeating them with the raw 11-point ideology scores (see section S9.5). (F) Last, our data proved compatible with the broader body of research on the predictors of online news exposure (*6*). Political interest and ideology were specifically predictive of the amount of political online news exposure and, in particular, longer visits of political online news articles (section S9.6).

## Supplementary Materials

### This PDF file includes:

Sections S1 to S9 Tables S1 to S14 Figs. S1 to S19 References
