# Supplementary material for: Ideological self-selection in online news exposure: Evidence from Europe and the US
Source: Sci Adv. 2024 Sep 13;10(37):eadg9287. doi: 10.1126/sciadv.adg9287 (PMC11397479; doi:10.1126/sciadv.adg9287)
Supplement: Supplementary file 1 — Sections S1 to S9 Tables S1 to S14 Figs. S1 to S19 References [file sciadv.adg9287_sm.pdf]

Science Advances
AAAS

Supplementary Materials for

Ideological self-selection in online news exposure: Evidence from Europe and the US

Frank Mangold *et al*.

Corresponding author: Frank Mangold, frank.mangold@gesis.org

Sci. Adv. **10**, eadg9287 (2024)
DOI: 10.1126/sciadv.adg9287

This PDF file includes:

Sections S1 to S9
Tables S1 to S14
Figs. S1 to S19
References

S1 Sample composition and country coverage

Data was collected from online access panels of the market research company *Netquest* whose participants consented to regularly participate in surveys and install tools tracking their browsing behavior on desktop computers and/or mobile devices. Participants can temporarily pause the tracking tools at any time. In countries (France, Spain, US) where the web tracking panels had a sufficient size, approximately 1,500 participants were invited according to population margins, but some quota cells still remained empty. In countries where the panels did not have a sufficient size, all panelists were invited (Germany, Italy, UK).

Compared to national population margins, the samples are skewed towards well-educated female middle-aged people (Table S1). Especially younger and older people as well as the lower educated strata of the population are underrepresented. At the same time, especially elderly and lower educated people generally use the Internet less. Unfortunately, high-quality benchmark data on the demographics of Internet users are not available for each country.

Table S1: Demographics by Country (%).

	Gender	Age					Education*		
Country	Female	29/under	30-39	40-49	50-59	60/over	Low	Medium	High
France	55.00	14.84	19.67	22.02	21.88	21.60	4.69	51.48	43.82
Germany	51.27	13.29	19.32	20.55	27.33	19.51	28.28	37.70	34.02
Italy	57.84	14.49	25.24	28.64	19.63	12.00	10.26	46.74	43.00
Spain	51.52	20.97	10.56	15.99	22.53	29.96	24.09	33.46	42.45
UK	52.29	9.72	15.32	20.64	23.85	30.46	4.95	48.53	46.51
US	65.56	13.04	20.75	18.52	23.85	23.85	4.47	60.59	34.94

Note: *Harmonized based on the International Standard Classification of Education (ISCED).

Table S2: Country-Level Characteristics

Country	Party system	Media system
France	Multi-party	Polarized pluralist (+ reduced ideological selectivity)
Germany	Multi-party	Democratic corporatist (+ relatively weak public broadcasting online)
Italy	Multi-party	Polarized pluralist
Spain	Multi-party	Polarized pluralist
UK	Two-party	Liberal (+ strong public broadcasting online)
US	Two-party	Liberal (+ strongest proliferation of partisan media and potential drift towards "polarized")

Note: Characteristics in parentheses are country specifics that separate the countries from other media systems of the same category (*21, 34, 43, 7, 24, 6*).

S2 Survey items

Table S3: Survey Items Used in the Analysis

Variable	Description	Original source
Age	Self-reported age in years.	European Social Survey, Round 8 (ESS) (64)
Education	Country-specific education levels that were recoded into "low", "medium" and "high education" based on the country-comparative ISCED scheme.	ESS
Gender	Self-reported gender. Female was coded as 1, male and the few "other gender" responses as 0.	ESS
Political ideology	Ideological self-placement on 11-point scale ranging from 1 (left) to 11 (right). The end points in the US survey were labeled "very liberal" and "very conservative". Recoded into "left" (i.e., raw values from 1 to 5), "centrist", and "right" (i.e., raw values from 7 to 11) for methodological reasons and according to procedure outlined in the main paper.	ESS
Political extremism	Based on the political ideology question ranging from 1 (left) to 11 (right). Political extremism is calculated as the absolute distance of an individuals' ideology to the country mean (see also (65)) to control for gradual differences.	ESS
Political interest	Measured as a 4-point scale ranging from "not at all interested" to "very interested".	ESS

S3 News domain coding

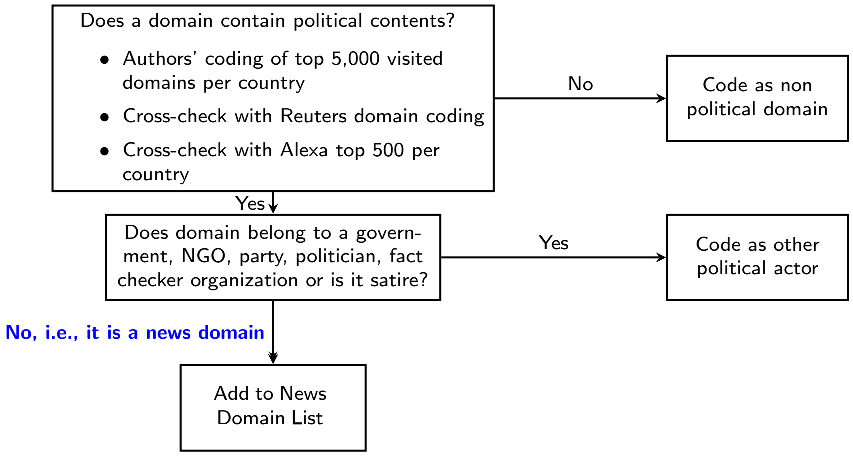

Figure S1: Summary of the News Domain Coding.

S4 Descriptive statistics

Table S4: Descriptive Statistics, France

Variable	N	Mean	St. Dev.	Min	Median	Max
Survey variables						
Age	1,444	46.29	14.28	18.0	47.0	85.00
Education	1,444	2.39	0.58	1.0	2.00	3.00
Female	1,444	0.55	0.50	0.0	1.00	1.00
Political interest	1,443	2.73	0.91	1.0	3.00	4.00
Political ideology	1,444	6.10	2.60	1.00	6.00	11.00
Political extremism	1,440	1.93	1.75	0.10	1.90	5.10
News visits						
Total news visits	1,444	261.25	718.16	0.00	80.00	20,534.00
News outlets visited	1,444	11.94	10.03	0.00	10.00	68.00
Political news visits	1,444	24.74	86.29	0.00	4.00	1,947.00
Intermediary visits						
Facebook visits	1,444	1,883.17	3,831.09	0.00	325.00	38,434.00
Twitter visits	1,444	93.55	720.07	0.00	1.00	16,938.00
Search visits	1,444	2,485.32	4,463.77	0.00	1,085.00	58,869.00
Portal visits	1,444	1,519.05	3,652.09	0.00	41.00	40,043.00
Total visits	1,444	21,402.00	28,054.02	1.00	14,669.00	546,651.00

Table S5: Descriptive Statistics, Germany

Variable	N	Mean	St. Dev.	Min	Median	Max
Survey variables						
Age	1,055	46.94	14.05	18.00	48.00	84.00
Education	1,055	2.06	0.79	1.00	2.00	3.00
Female	1,055	0.51	0.50	0.00	1.00	1.00
Political interest	1,052	2.87	0.86	1.00	3.00	4.00
Political ideology	1,055	5.64	2.00	1.00	6.00	11.00
Political extremism	1,055	1.49	1.34	0.36	1.36	5.36
News visits						
Total news visits	1,055	237.71	826.40	0.00	32.00	17,300.00
News outlets visited	1,055	9.04	10.17	0.00	6.00	63.00
Political news visits	1,055	28.23	172.23	0.00	1.00	4,511.00
Intermediary visits						
Facebook visits	1,055	860.95	2,366.45	0.00	58.00	21,703.00
Twitter visits	1,055	41.81	584.75	0.00	0.00	17,975.00
Search visits	1,055	1,599.27	3,434.47	0.00	571.00	35,350.00
Portal visits	1,055	1,466.92	3,298.31	0.00	135.00	50,790.00
Total visits	1,055	16,238.93	26,029.12	1.00	8,956.00	395,178.00

Table S6: Descriptive Statistics, Italy

Variable	N	Mean	St. Dev.	Min	Median	Max
Survey variables						
Age	1,436	43.78	12.82	18.00	43.00	88.00
Education	1,436	2.33	0.65	1.00	2.00	3.00
Female	1,436	0.58	0.49	0.00	1.00	1.00
Political interest	1,434	2.74	0.84	1.00	3.00	4
Political ideology	1,431	6.29	2.76	1.00	6.00	11.00
Political extremism	1,431	2.20	1.68	0.28	2.28	5.28
News visits						
Total news visits	1,436	245.57	601.64	0.00	50.00	7,554.00
News outlets visited	1,436	9.87	9.68	0.00	7.00	92.00
Political news visits	1,436	16.42	65.24	0.00	1.00	1,390.00
Intermediary visits						
Facebook visits	1,436	1,990.14	4,453.10	0.00	372.50	55,557.00
Twitter visits	1,436	53.71	432.68	0.00	0.00	10,453.00
Search visits	1,436	2,842.27	4,568.30	0.00	1,327.50	58,632.00
Portal visits	1,436	1,085.51	2,579.29	0.00	59.50	45,743.00
Total visits	1,436	17,726.12	21,733.47	1.00	10,794.00	309,172.00

Table S7: Descriptive Statistics, Spain

Variable	N	Mean	St. Dev.	Min	Median	Max
Survey variables						
Age	1,342	48.49	16.75	18.00	50.00	87.00
Education	1,342	2.18	0.79	1.00	2.00	3.00
Female	1,342	0.52	0.50	0.00	1.00	1.00
Political interest	1,341	2.72	0.83	1.00	3.00	4.00
Political ideology	1,342	4.96	2.67	1.00	5.00	11.00
Political extremism	1,342	2.23	1.47	0.04	1.91	6.04
News visits						
Total news visits	1,342	347.17	745.88	0.00	84.00	9,847.00
News outlets visited	1,342	14.37	11.96	0.00	12.00	69.00
Political news visits	1,342	41.92	134.49	0.00	5.00	2,050.00
Intermediary visits						
Facebook visits	1,342	1,037.15	2,705.49	0.00	134.00	39,501.00
Twitter visits	1,342	188.57	1,202.70	0.00	3.00	24,942.00
Search visits	1,342	2,464.94	3,065.75	0.00	1,385.00	27,519.00
Portal visits	1,342	202.00	974.70	0.00	3.00	26,717.00
Total visits	1,342	13,121.04	15,908.53	1.00	8,927.00	259,844.00

Table S8: Descriptive Statistics, UK

Variable	N	Mean	St. Dev.	Min	Median	Max
Survey variables						
Age	1,090	50.66	14.90	18.00	51.00	89.00
Education	1,090	2.42	0.59	1.00	2.00	3.00
Female	1,090	0.52	0.50	0.00	1.00	1.00
Political interest	1,089	2.72	0.91	1.00	3.00	4.00
Political ideology	981	6.00	1.96	1.00	6.00	11.00
Political extremism	981	1.34	1.44	0.00	0.96	5.00
News visits						
Total news visits	1,090	489.50	1,075.48	0.00	110.00	11,671.00
News outlets visited	1,090	9.65	9.18	0.00	7.00	78.00
Political news visits	1,090	43.18	164.70	0.00	2.00	2,773.00
Intermediary visits						
Facebook visits	1,090	1,461.71	3,118.28	0.00	189.00	36,498.00
Twitter visits	1,090	204.72	1,475.74	0.00	2.00	31,759.00
Search visits	1,090	2,692.34	5,358.99	0.00	876.50	74,496.00
Portal visits	1,090	1,153.82	3,261.83	0.00	42.00	40,032.00
Total visits	1,090	19,098.78	20,572.97	1.00	12,474.00	162,615.00

Table S9: Descriptive Statistics, US

Variable	N	Mean	St. Dev.	Min	Median	Max
Survey variables						
Age	1,387	47.54	14.80	18.00	48.00	85.00
Education	1,387	2.30	0.55	1.00	2.00	3.00
Female	1,387	0.66	0.48	0.00	1.00	1.00
Political interest	1,386	2.64	0.97	1.00	3.00	4.00
Political ideology	1,382	6.44	2.72	1.00	6.00	11.00
Political extremism	1,382	2.14	1.69	0.44	1.54	5.44
News visits						
Total news visits	1,387	170.56	564.40	0.00	24.00	12,535.00
News outlets visited	1,387	9.27	11.50	0.00	5.00	121.00
Political news visits	1,387	25.29	126.60	0.00	1.00	2,839.00
Intermediary visits						
Facebook visits	1,387	2,038.31	4,701.19	0.00	426.00	108,213.00
Twitter visits	1,387	149.01	1,235.67	0.00	2.00	30,306.00
Search visits	1,387	3,596.66	5,985.36	0.00	1,387.00	72,289.00
Portal visits	1,387	1,646.14	3,635.10	0.00	125.00	41,657.00
Total visits	1,387	21,896.21	30,961.52	1.00	13,663.50	499,258.00

Figure S2: Ideological Alignment Scores of Top 15 News Websites (in % Reach) per Country

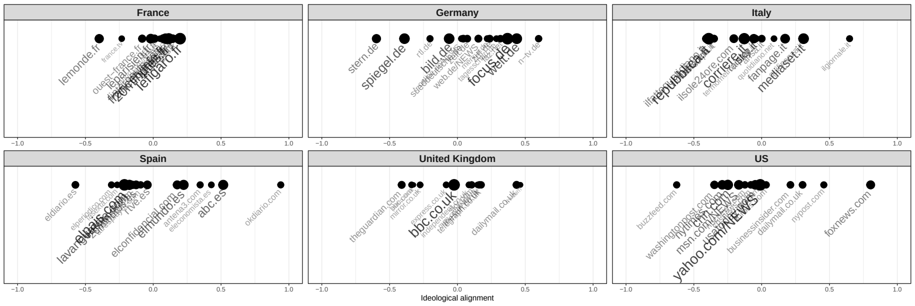

Note: Points are individual news outlets, with point size being proportional to % reach. Additional scores for the US in Figure S13.

S5 Comparison of samples with external benchmarks

To assess the generalizability of the news consumption behavior of study participants, we compare the popularity of news domains in our data to their visit numbers in the top 500 Alexa country rankings for the three months of our data collection (https://www.alexa.com/siteinfo). Alexa has the advantage that the data is available across countries, as it tracks the website visits of more than 300 million users who have installed a web browser plugin. Nevertheless, it is still unclear how representative of each countries' online population the data is. Figure S3 shows the correspondence between the number of news website visits in both data sources.

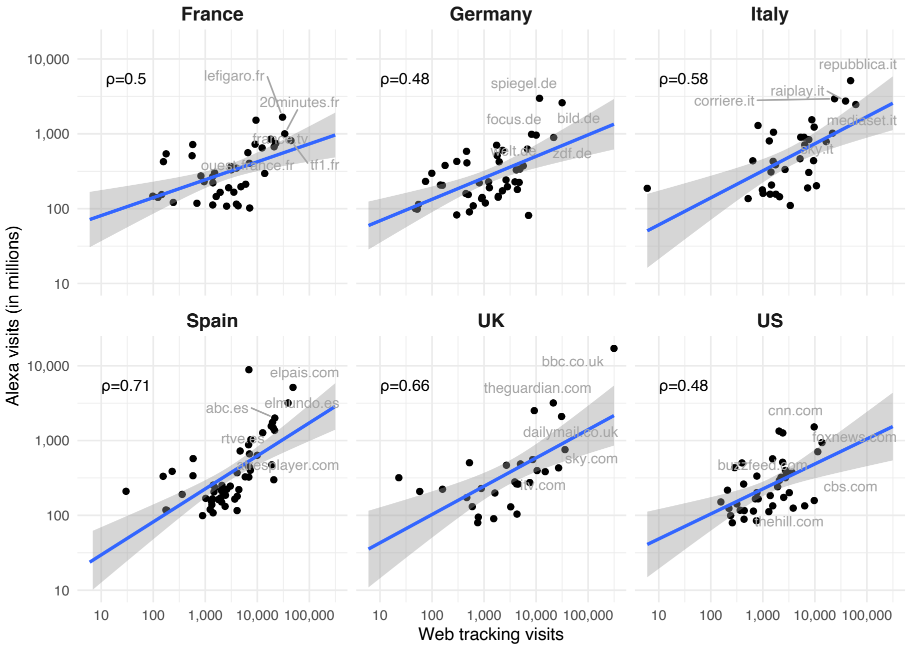

Figure S3: Popularity of news websites in the top 500 Alexa rankings per country and among web tracking panelists. ρ = Spearman's rank correlations.

Participants in an online web tracking might have a higher propensity to get news from online media instead of newspapers, television, and radio. To compare offline news exposure to an external benchmark, we implemented self-report items of media exposure from the Reuters Digital News Report (DNR) 2019 in our surveys (*66*). The high correlations demonstrate that the study participants were equally likely to get news from newspapers and in particular from the major television news programs in each country.

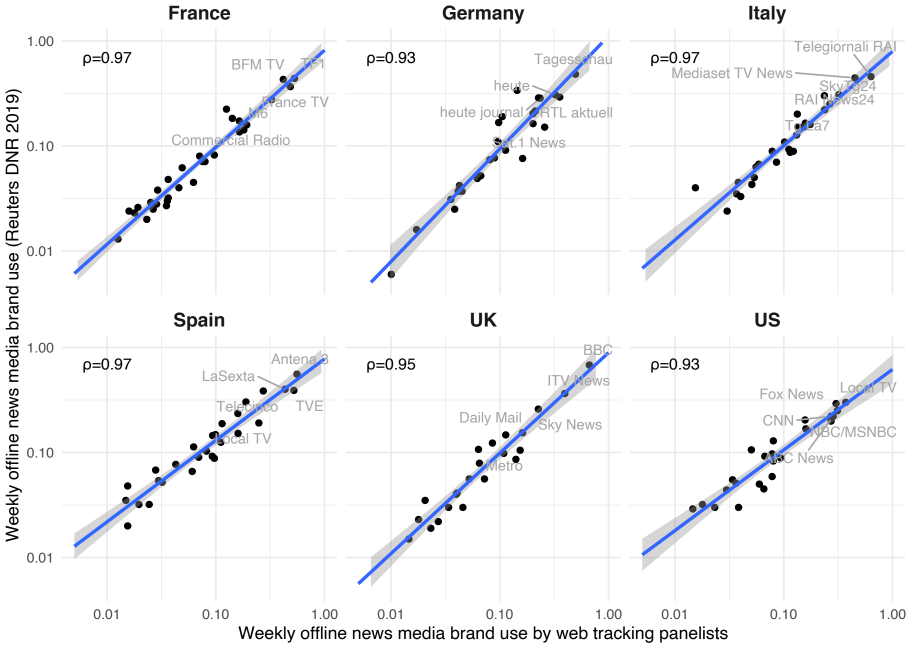

Figure S4: Weekly offline news media brands used, comparison of Reuters Digital News Report 2019 and web tracking panelists. ρ = Spearman's rank correlations.

Another important way in which the study participants could differ from other online news users might be privacy attitudes. We therefore investigated to what extent privacy attitudes of web tracking panelists diverged from panelists who participate in surveys, but do not have tracking tools installed (replicating the approach of (*29*)). As a comparison group, we sampled 1,002 German participants based on population margins for gender, age and education from the regular online access panel of the same survey company. Respondents were presented the following statements and asked about their (dis)agreement on a five-point scale.

- Personalized advertising makes me afraid.
- I am concerned about how much data there is about me on the Internet.
- My privacy on the Internet does not matter to me.

Figure S5 shows that there were only marginal differences in the privacy attitudes of online access panelists who participated in the web tracking and those who did not. Yet as outlined in Section S1, we cannot draw inferences to the privacy attitudes of the German general population from these data.

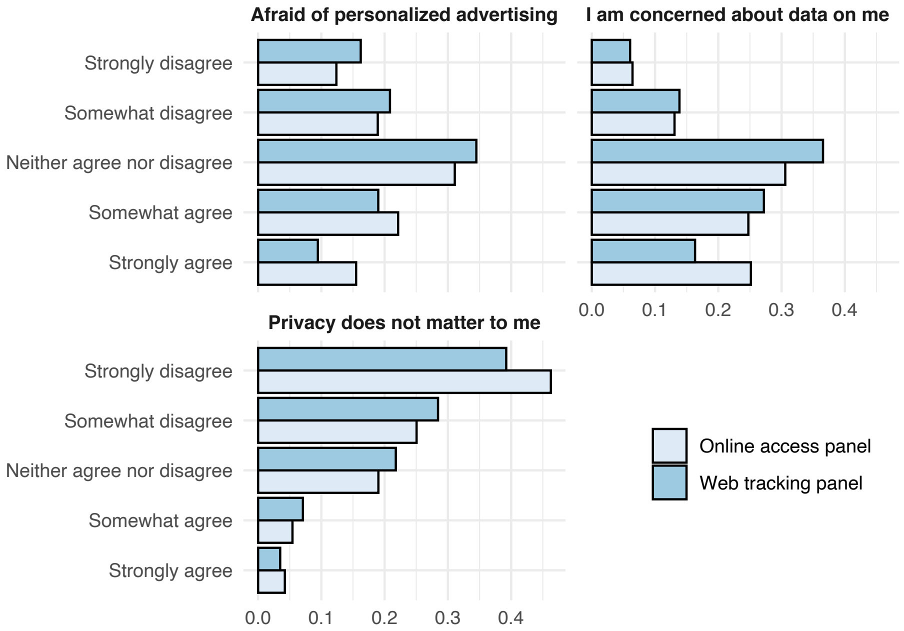

Figure S5: Privacy attitudes among German web tracking participants and a sample of German online access panelists without web tracking.

S6 Classifying political news articles

We build on previous work combining web tracking data and article content (*51,28,29*) by constructing a classifier for each country that predicts whether the news articles visited by panelists contain political content. To get the textual contents, all unique URLs were crawled with the R package *rvest* (*67*). The article text was parsed from the downloaded html files by means of the Python library *newspaper* (*68*).

For the purposes of training the text classification model, we first selected five major news websites in each country and identified all of their articles that contain one of the unambiguous political keywords `polit`, `democrac`, or `elect` in the URL (see Table S10). We defined all articles as political whose content is related to either polity (e.g., political institutions, democracy), politics (e.g., elections, political actors, scandals) or policy (e.g., regulation or legislation with regard to substantive issues, which excludes nonpolicy aspects like crime reports). The respective five news outlets per country were chosen based on two considerations: (1) they are popular among our panelists and the overall online population according to the Reuters Digital News Report (*66*), and (2) they have a website/URL architecture with a specific `politics` subsection.

Table S10: Selected News Domains and Political Keywords per Country

Country	Outlets	Detected keywords in URLs
France	lefigaro.fr, 20minutes.fr, lemonde.fr, francetvinfo.fr, lepoint.fr	polit, democra, elections
Germany	bild.de, welt.de, focus.de, spiegel.de, sueddeutsche.de	polit, demokrat, wahl
Italy	repubblica.it, corriere.it, mediaset.it, leggo.it, ilmessaggero.it	polit, democraz, elezion
Spain	elpais.com, lavanguardia.com, elperiodico.com, eldiario.es, cadenaser.com	polit, democra, elecciones
UK	bbc.co.uk, theguardian.com, telegraph.co.uk, mirror.co.uk, independent.co.uk	polit, policy, democrac, elect
US	cnn.com, foxnews.com, nytimes.com, washingtonpost.com, nbcnews.com	polit, policy, democrac, elect

We treated the URLs of the five selected news domains that do not include one of the political keywords as the negative set of training articles. This is a restrictive operationalization of political news, as URLs on other sections of these websites also contain political content. Guess (*29*) and Flaxman et al. (*28*), in contrast, used a more extensive training dataset including URLs published on website subsections such as `business`, `national` or `news`. While our classifier leads to more conservative estimates of the share of political content, its parsimony aids the cross-national comparability of results. Descriptive statistics of the amount of online news exposure classified as political in line with our methodological premises are provided in Table S14.

The text analysis was performed using the R package *quanteda* (*69*). The following text preprocessing steps were taken before training the classifiers:

1. We excluded the top level news domains (e.g., nytimes.com), as the content on these pages changes dynamically and therefore differed at the time of crawling from the time of the actual website visit made by a panelist.
2. As the included news domains publish in five different languages, we removed English, French (keeping the string "eu"), German, Italian and Spanish stopwords.
3. We removed punctuation, numbers, hyphens and symbols.
4. We reduced the corpus for each country to words that occur at least 20 times and construct unigrams as input features for the classifier.

The frequencies of words in these pre-processed corpora already reveal a clear signal: political articles have a distinct vocabulary compared with nonpolitical articles (Figure S6).

Using the articles including the URL keywords in Table S10 as "gold standard" labels for political news coverage, a Naive Bayes classifier was trained for each country and evaluated against a held-out set of test data using ten-fold cross-validation. The average performance of each Naive Bayes classifier per country across its respective ten folds is listed in Table S11. The classifiers accurately identified political articles, mirroring the performance achieved in similar applications (*28, 29*). Table S12 shows the most predictive features for classifying articles as political or nonpolitical.

Table S11: Results from Ten-Fold Cross-Validation

Country	Accuracy	Precision	Recall	F1
France	0.92	0.92	0.99	0.96
Germany	0.91	0.91	0.98	0.95
Italy	0.93	0.93	0.99	0.96
Spain	0.89	0.88	0.98	0.93
UK	0.94	0.95	0.99	0.97
US	0.88	0.89	0.96	0.92

Note: Mean values from ten-fold cross-validation.

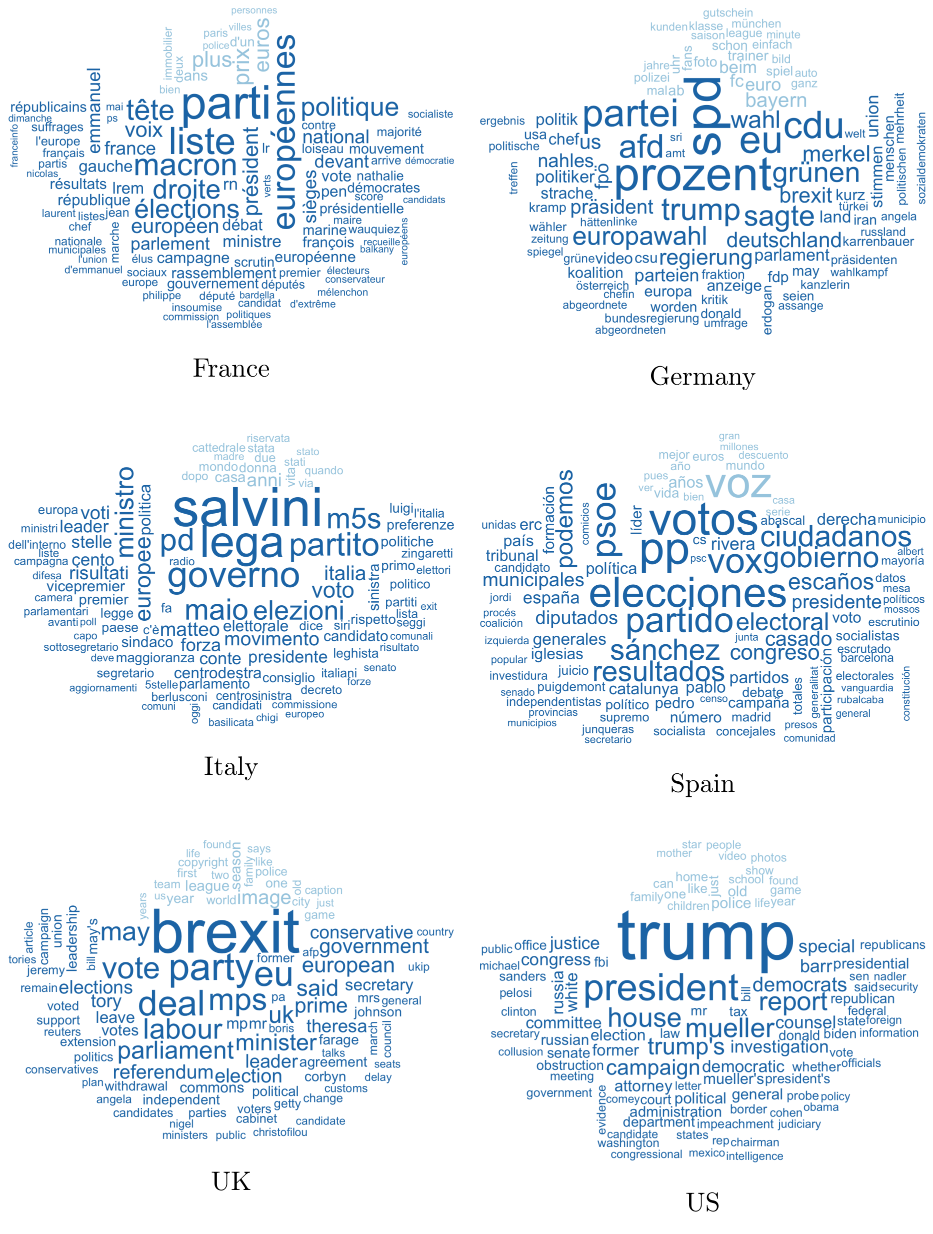

Figure S6: Wordclouds with most frequent words in political articles (bottom of each figure) vs. nonpolitical articles (top of each figure) on the websites of five major news outlets per country.

After the evaluation, the classifier was applied to all news articles that were visited by the study participants to predict whether these are political or not. A validation of the classifier predictions by hand-coding 100 randomly selected articles for each country showed a highly accurate out-of-sample performance. For constructing the final measure identifying political articles, we first applied the political URL keywords listed in Table S10 to all URLs and only used the classifier predictions if there was no positive string match. Above and beyond the 113,420 news website visits classified as political by the URL keywords, we identified an additional number of 178,969 visits to political articles (e.g., on website sections such as `national` or `society`) thanks to the classifier.

Table S12: Most Predictive Features for Classifying Political vs. Nonpolitical Articles

Country	Political	Nonpolitical
France	plus, c'est, macron, liste, france, aussi, président, comme, fait, parti, européennes, qu'il, politique, emmanuel, d'un, tout, d'une, être, faire, deux	plus, c'est, d'un, deux, ans, aussi, comme, d'une, fait, tout, france, après, euros, bien, être, paris, selon, qu'il, prix, faire
Germany	prozent, spd, mehr, sagte, eu, partei, deutschland, cdu, afd, wurde, trump, anzeige, menschen, grünen, seit, europawahl, wahl, zwei, schon, lesen	mehr, wurde, schon, euro, zwei, immer, lesen, ab, gibt, beim, jahren, deutschland, seit, geht, anzeige, mal, menschen, sagte, drei, bild
Italy	salvini, lega, governo, pd, partito, stato, m5s, ministro, maio, poi, presidente, dopo, fa, solo, italia, prima, elezioni, due, c'è, essere	anni, stato, dopo, due, prima, essere, poi, solo, quando, fatto, ancora, stata, sempre, così, casa, fa, via, euro, molto, fare
Spain	pp, partido, gobierno, elecciones, vox, psoe, votos, sánchez, ciudadanos, dos, podemos, españa, país, electoral, ser, madrid, presidente, casado, años, tras	voz, años, dos, ser, puede, así, hace, ahora, españa, según, vez, euros, solo, después, madrid, tres, día, además, cada, año
UK	brexit, said, party, deal, may, eu, vote, mps, uk, labour, people, parliament, minister, government, mr, one, european, prime, getty, new	said, image, one, people, first, year, time, can, two, new, just, caption, getty, us, years, now, says, copyright, like, last
US	trump, said, president, house, report, mueller, trump's, one, news, democrats, new, campaign, people, justice, told, fox, us, white, barr, investigation	said, one, people, new, like, just, time, year, can, told, get, news, first, two, years, now, trump, according, day, even

S7 Description of statistical analysis

This section provides background information on the descriptive statistics and multilevel results reported in the main paper. Further background on the multilevel results is provided in Section S8. Further conceptual and statistical background of the descriptive measures' relations to the ones used in the robustness tests is provided in Section S9.2.

Descriptive statistics

The study of Simpson's D (*62*) follows the conceptual understanding of diversity as a multiplex two-fold concept. It goes beyond the more simplistic questions of how many outlets users visit or whether their online news diets comprise visits to outlets of different categories (like more liberal, centrist, and conservative news outlets). It integrates more gradual differences in online news diet composition by additionally considering news diet balance or, alternatively speaking, how equally users' news visits are distributed across categories (*16*).

Meanwhile, the calculation of ideological news diet diversity comes with the specific challenge that it requires categorizing news outlets as liberal, centrist, and conservative. To this end, we followed prior research that has relied on alignment scores and used them to implement relatively lenient operational definitions of what counts as liberal and conservative (*16, 49*). Specifically and in light of the theoretical range of the alignment scores from -1 to 1 (which is determined by recoding participants' ideological self-placement to values -1 to 1, as outlined in the main paper, and also practically restricts the standard deviation of online news diet slant to a range from 0 to 1), we categorized outlets with scores of above 0.20 as more right-leaning, outlets with scores of less than –0.20 as more left-leaning, and outlets with scores between -0.20 and 0.20 as more centrist. While the choice of any threshold is somewhat arbitrary, the main advantage of our categorization scheme is that it sets the bar for categorizing news visits as cross-cutting online news exposure fairly low. This aids the substantive interpretability of the obtained online news diet diversity scores such that low numerical scores in this metric specifically rule out cross-cutting online news exposure.

Finally, the standard deviation (SD) of online news diet slant is directly related yet not mathematically identical to the "expected squared distance" (ESD) derived by (*28*), since the ESD is mathematically equivalent to multiplying the SD with the square root of two. Against this backdrop, we empirically demonstrate in Section S9.2 that the difference between the statistics does not lead to other substantive results on the differences between political and nonpolitical news, temporal thresholds, and countries.

Multilevel regression analysis

The multilevel approach used in the multivariate analyses of ideological selectivity takes into account that individual news visits (i.e., level-1 units of analysis)

are nested within participants (i.e., level-2 units) which are, in turn, nested within countries (level-3 units). Due to the relatively low number of country samples, country differences were modeled by means of fixed rather than random effects. This comes with the inherent advantage that the estimates for the person-level characteristics (like political interest) and news access exclusively mirror empirical differences within countries, both in line with the theoretical rationale in the main paper and safeguarding inferences against biases due to (previously) unmeasured country-level confounders (*70, 55*). The basic multilevel model is defined as:

$$y_{ijk} = \mu + \beta_1 I_{jk} + \beta_2 C_k + \beta_3 P_{jk} + \beta_3 A_{ijk} + \epsilon_{ij0}$$

where y_{ijk} refers to the ideological alignment associated with the outlet of each visit i by participant j from country k; I_{jk} to the participant's ideological leaning; C_k to the country differences; P_{jk} to the set of person-level covariates; and A_{ijk} to the access modes for the individual news visits. For the purposes of illustration, we refrained from formally representing categorical predictors by separate terms. We extended the basic multilevel model by calculating the interactions of ideological leaning with countries, person-level characteristics, and news access modes and used the resulting interactions terms to determine the conditional differences between the online news diet slant of liberals and conservatives reported in Figure 4 in the main paper as well as Figure S7 in the SM. The corresponding confidence intervals for the conditional differences signify whether the respective difference between liberals and conservatives was significantly greater than zero. Figure S8 plots the underlying interaction terms, whose confidence intervals provide direct tests of the differences in the conditional differences (which we took into account in the interpretations made in the main paper). How the corresponding regression coefficients statistically correspond to the each other is explained in greater technical detail in the following section.

On more general notes, the multilevel approach establishes compatibility with prior research because within the 3-level framework taken, ideology is a level-2 (i.e., person-level) predictor. Consequently, the resulting regression coefficients are conceptually equivalent to the regression estimates produced in prior research that has first calculated the overall ideological lean ("slant") of each respondent's media diet (by averaging over the alignment scores for the domains associated with her news visits) and subsequently regressed the obtained aggregated (mean) scores on person-level correlates (like demographics, political interest, etc.). Meanwhile, the multilevel approach better preserves the granularity in the data and thereby allows to, for instance, directly take into account news access modes on the visit-level. It allows for thorough assessments across different levels of analysis while simultaneously accounting for the differences in the individual participants' online news diets in a way that both enables statistical contrasts across news diets of different sizes and prevents that heavy users with an exceptionally large number of visits exert an over-proportional influence on the regression estimates obtained (*55*).

S8 Supplementary regression outputs

Figure S7 complements Figure 4 in the main paper by additionally covering the controls: political extremity, age, gender, and education. To facilitate the interpretation, we rescaled the age variable in a way that the estimates reflect the generational difference in ideological selectivity between an average millennial (born 1989) and an average boomer (born 1954). Figure S8 summarizes the interactions between ideology with countries, person-level characteristics, and news access modes estimated for the methodological reasons outlined in the Section S7. Here, confidence intervals that do not contain the value zero signify that the respective conditional differences reported in Figure S7 were significantly different. For instance, if we look at Figure S8, Column F, we see that compared to women (i.e., the reference category), the political online news exposure of men exhibited significantly higher ideological selectivity across all temporal thresholds. This corresponds to the, though not extremely, generally higher values for men in Figure S7, Column F.

Beyond the results reported in the main paper, the robustness test strengthened the confidence in our main results. The political online news exposure of men showed higher ideological selectivity across all temporal thresholds. By contrast, the higher ideological selectivity of older participants was more confined to the longer thresholds, indicating that they might lack the digital media literacy for bringing their news exposure more in line with their ideology from the outset (for related results and arguments, see (*29, 71*)). Education did not have a significant impact on ideological selectivity.

Figure S7: Conditional Differences in Liberals and Conservatives' Online News Diet Slant (Full Output)

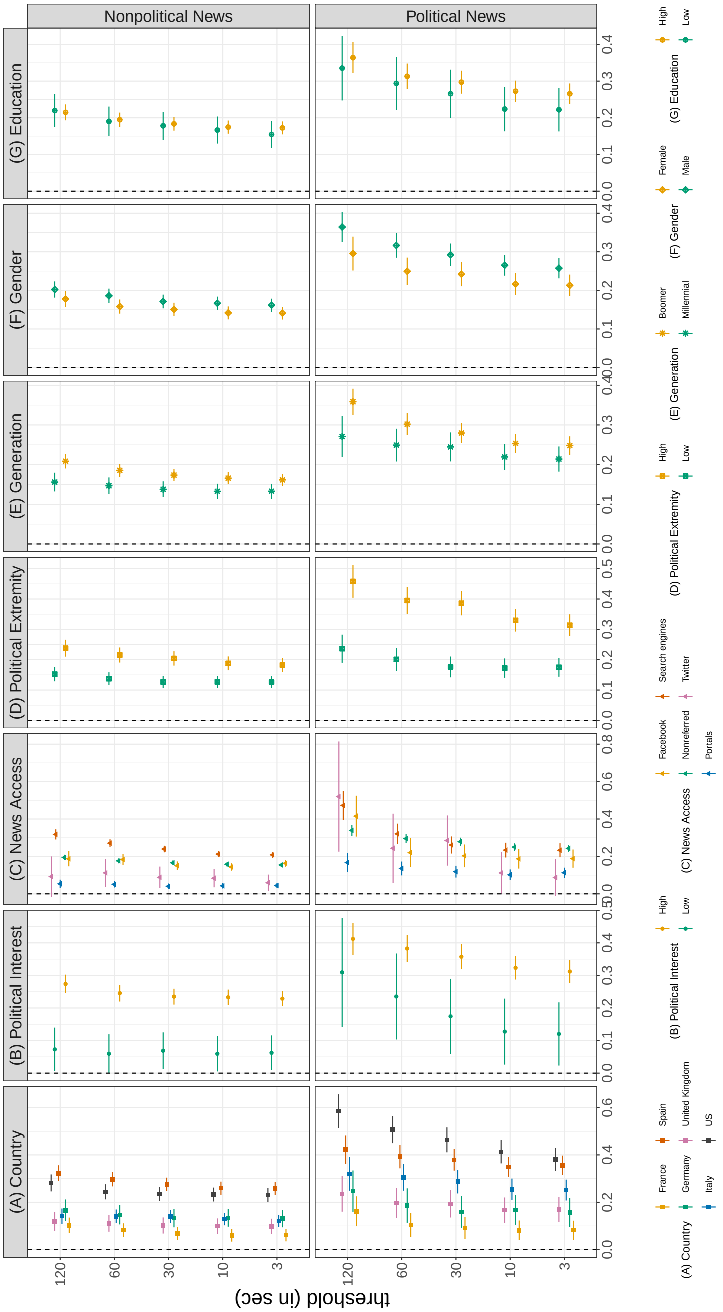

Note: Regression coefficients and 95% confidence intervals from multilevel regression models. The figure complements Figure 4 in the main paper and is explained within the text.

Figure S8: Interactions of Political Ideology with Macro- and Micro-Level Correlates

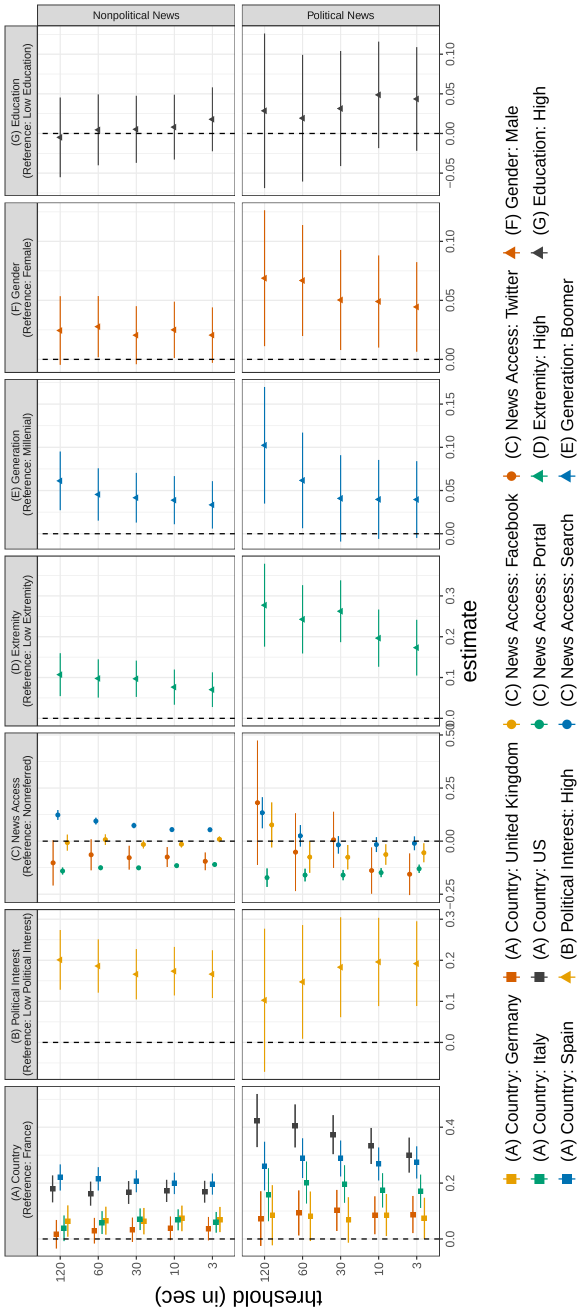

Note: Regression coefficients and 95% confidence intervals from multilevel regression models. The figure complements Figure S7 and is explained within the text. The reference categories were "(A) Country: France", "(B) Political Interest: Low", "News Access: Nonreferred", (D) Political Extremity: Low", "(E) Generation: Millennial", "(F) Gender: Female", and "(G) Education: Low."

S9 Robustness tests

S9.1 Prevalence of online news exposure

This section reports statistics that complement the results on the prevalence of online news exposure in the main paper. To this end, Table S13 summarizes the high-level audience metrics verbally described at the beginning of the results section. We see that despite some variation between countries, news in general accounted for a small fraction of total visits within each country. More than 85% of participants in each country visited a news domain at least once during the 3-month period covered by our study.

Table S13: Fraction of Website Visits to News Articles and News Visitors

country	all website visits	news visits %	news visitors %
France	31,011,502	1.22%	91.93%
Germany	17,229,504	1.46%	86.62%
Italy	25,561,072	1.39%	90.64%
Spain	17,647,797	2.65%	94.05%
United Kingdom	20,817,668	2.56%	92.57%
US	30,391,944	0.78%	86.67%

Table S14 breaks down the fraction on news visits classified as news use episodes with each of the temporal thresholds as well as the proportions of visits to political and nonpolitical news per temporal threshold. We see that the longer thresholds naturally decreased the total number of news episodes substantively. While political news accounted for less than 20% of news episodes with all thresholds, the share of political news episodes increased with the longer thresholds in each country. This means that the strong drop of the share of political news visitors reported in Figure 1 in the main paper was not an artifact of participants spending shorter periods on political than nonpolitical news articles. More generally, it indicated that while political news visits were overall less prevalent and more strongly concentrated on a narrower fraction of the online audience than nonpolitical news visits, those visitors who spend longer periods of time on political news articles also devoted a disproportionately high volume of their online news diets to political news. Notably, the estimates in Table S14 also matched the methodological tenets outlined in the Section S6. While highest among our set of countries, the share of political news visits for the US generally remained smaller than reported in (*29*) (19% in 2015, 23% in 2016). The difference may be partly explained by the ongoing presidential election in his 2016 study (which most likely increased exposure to political news) yet was also in line with our premise that our classifier produces more conservative estimates of the share of political content.

Table S14: Summary Statistics for News Visits

Country		3 sec	10 sec	30 sec	60 sec	120 sec
France	News visits	302,867	225,546	137,021	82,366	40,716
	in %	(100.0)	74.5	45.2	27.2	13.4
	nonpol/pol	91/9	90/10	88/12	87/13	87/13
Germany	News visits	270,769	198,508	124,360	76,576	38,916
	in %	(100.0)	73.3	45.9	28.3	14.4
	nonpol/pol	90/10	91/11	87/13	86/14	86/14
Italy	News visits	277,835	198,372	118,968	71,541	34,253
	in %	(100.0)	71.4	42.8	25.7	12.3
	nonpol/pol	94/6	92/8	91/9	91/9	91/9
Spain	News visits	468,821	347,788	223,466	146,143	77,520
	in %	(100.0)	74.2	47.7	31.2	16.5
	nonpol/pol	87/13	86/14	85/15	84/16	83/17
United Kingdom	News visits	469,324	334,212	194,032	118,097	62,588
	in %	(100.0)	71.2	41.3	25.2	13.3
	nonpol/pol	91/9	90/10	89/11	88/12	88/12
US	News visits	189,586	147,111	99,416	56,259	27,130
	in %	(100.0)	77.6	52.4	29.7	14.3
	nonpol/pol	85/15	85/15	84/16	84/16	83/17

S9.2 Descriptive analyses of ideological self-selection

The analyses reported in Figure 3, Row A, in the main paper followed research which has calculated the ideological segregation of news audiences in the form of the "isolation" index (*17, 5*). As a robustness check, we repeated these calculations using two other popular segregation indices: the dissimilarity index (*72*) and the Atkinson index (*73*). Following the notation used in the main paper, the dissimilarity index can be formally defined as

$$DS = \frac{1}{2} \sum_{i \in I} \frac{c_i}{c_{total}} - \frac{l_i}{l_{total}}$$

Likewise, the Atkinson index can be formally defined as

$$A = \sum_{i \in I} \left(\frac{c_i}{c_{total}} \right)^{\frac{1}{2}} \cdot \left(\frac{l_i}{l_{total}} \right)^{\frac{1}{2}}$$

In line with the isolation index, both the dissimilarity index and Atkinson index range from 0 to 1 and higher numerical values indicate relatively higher ideological segregation. The main conceptual difference among the three statistics exists between the dissimilarity index on the one hand and the isolation and Atkinson indices on the other hand. The dissimilarity index is less strongly centered on the question of whether liberal and conservatives have common exposure to news sources at all. Therefore, it takes relatively higher numerical values even when liberals and conservatives are not entirely separated in their news choices but some commonalities in these choices persist. This more gradual approach can be broken down to the simple question to what share of conservative (or liberal) news use would need to be redistributed across media for the share conservative to be uniform across outlets (see also (*17*)). The Atkinson index has been demonstrated to be the unique measure of segregation satisfying a set of important measurement axioms, including scale invariance, that enable particularly safeguarded comparisons of the relative differences between units of analysis (*74*). Looking at Figure S9, we see that despite these differences, all measures more or less perfectly agreed on the same relative differences in ideological segregation, including both the differences between political news and nonpolitical online news, temporal thresholds, and countries. The notable exception was a smaller relative difference of the US from the European countries produced by the dissimilarity index than by the isolation and Atkinson indices. The exception supported that —in line with the substantive conclusions drawn in the main paper—US partisans have more often abandoned the relatively few cross-cutting political online news experiences that have persisted in the European online news environments.

Figure S9: Atkinson Index, Dissimilarity Index, and Isolation Index

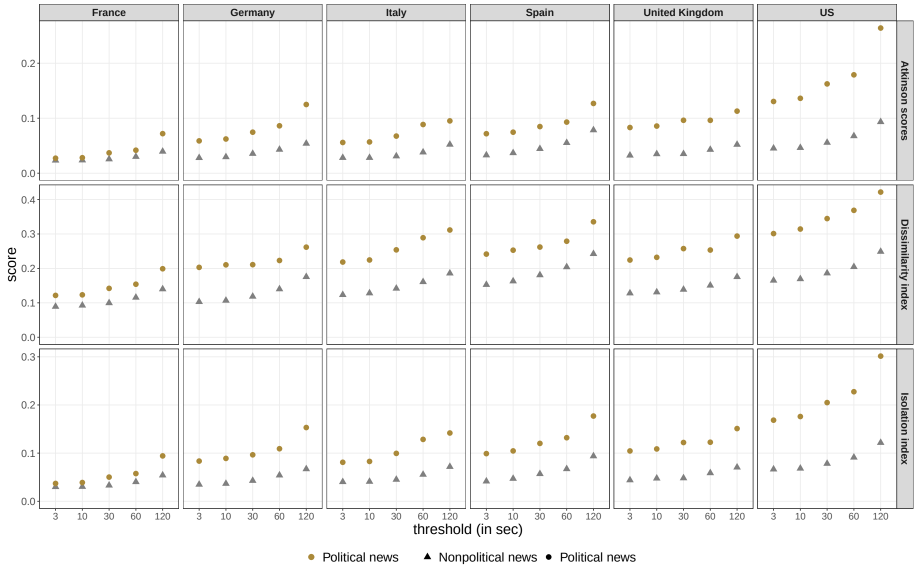

Note: The figure complements Figure 3, Row A, in the main paper by additionally reporting the ideological segregation scores obtained with the dissimilarity and Atkinson indices.

The analyses reported in Figure 3, Row B, rested on the calculation of the ideological diversity of online news diets by means of Simpson's D. As a robustness check, we repeated these calculations using another diversity measure that has undergone similarly rigorous validation in prior research, most notably because it is mathematically equivalent to what has also been called "entropy": Shannon's H (*75*). Following the notation used in the main paper, Shannon's H can be formally defined as:

$$H_j = -\sum_{c \in C} \frac{N_{cj}}{N_j} \; ln \frac{N_{cj}}{N_j}$$

While Shannon's H is less widely employed in audience research (exceptions are (*16*, *53*)) and is more difficult to interpret in absolute terms (because it is not restricted to numerical range zero to one), it allows for a similarly thorough investigation of the relative differences between news diets. Both Simpson's D and Shannon's H take into account the balance of participants' news visits across more conservative, centrist, and liberal news outlets. Meanwhile, Shannon's H puts a relatively stronger empirical emphasis on the question of whether news users' have at least a minimum of baseline exposure to both conservative, centrist, and liberal outlets at all (see also (*16*)). Looking at Figure S10, we see that both diversity measures nevertheless more or less perfectly agreed on the same relative differences, including the differences between political news and nonpolitical online news, temporal thresholds, and countries.

Figure S10: Shannon's H and Simpson's D

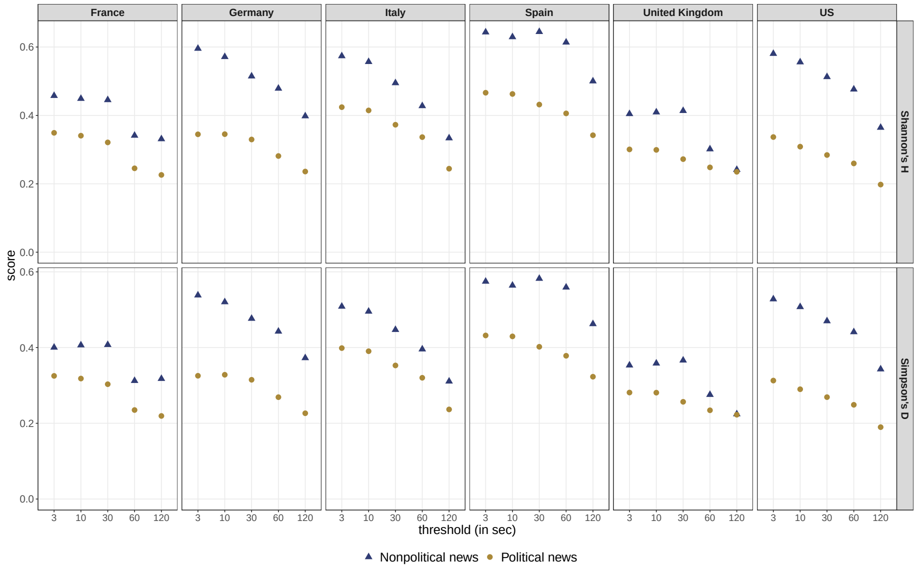

Note: The figure complements Figure 3, Row B, in the main paper by additionally reporting the (average) online news diet diversity obtained with Shannon's H.

As outlined in more mathematical terms in Section S7, the standard deviation (SD) of online news diet slant is related to the "expected squared distance" derived by (*28*). To establish that the difference between the statistics does not compromise the substantive results on the relative differences between both political and nonpolitical online news exposure, temporal thresholds, and countries, Figure S11 replicates and complements the SD of online news diet reported in Figure 3, Row C, in the main paper. It additionally reports the estimates obtained when calculating the expected squared distance between participants (in terms of them having left- vs. right-leaning news diets). While the expected squared distance yielded higher numerical values, both statistics agreed upon the relative differences between political and nonpolitical online news exposure, temporal thresholds, and countries. These commonalities notwithstanding, we urge readers to bear in mind that expected squared distances reported in Figure S11 are not directly comparable to the values reported in (*28*), because (A) these scholars operated with alignment scores with theoretical range from 0 to 1 (rather than —in our case—from -1 to 1) and, more fundamentally, (B) these scholars could not yet directly take into account the political leanings of online news users but used the IP addresses of news website visits to infer the counties where their visitors reside and subsequently used voting results from these counties to estimate the political composition of news outlets' usership.

Figure S11: Standard Deviation (SD) of Online News Diet Slant and Expected Squared Distance

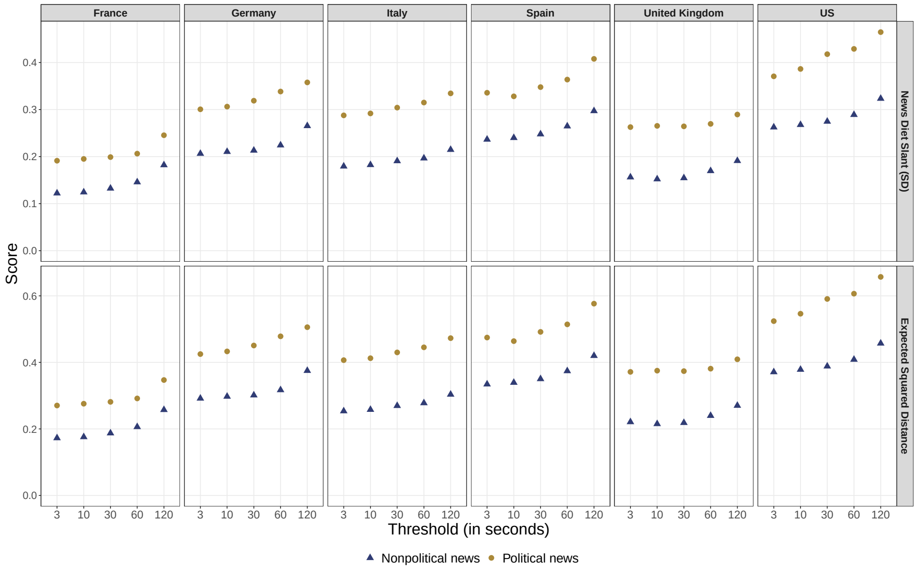

Note: The figure complements Figure 3, Row C, in the main paper by additionally reporting the expected squared distance between left- and right-leaning news diets.

Finally, Figure S12 complements Figure 2 in the main paper by plotting the distributions of liberals and conservatives' online news diet slant obtained with the 3-seconds threshold. The differences between political and nonpolitical online news exposure and countries exhibited similar tendencies yet were generally less pronounced compared to the differences obtained with the 120-seconds threshold. This finding further corroborated that when investigating online news exposure, it is important to consider both the differentiation between political vs. nonpolitical news and temporal thresholds.

Figure S12: Distributions of Online News Diet Slant of Liberals and Conservatives (3-Seconds Threshold)

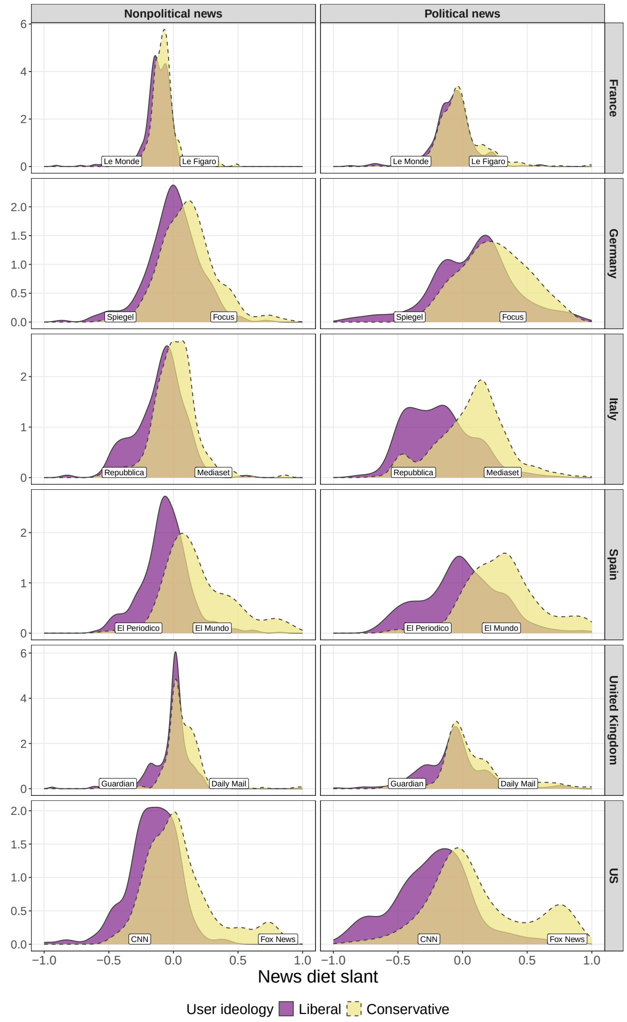

Note: The figure complements Figure 2 in the main paper by reporting the density distributions obtained with the 3-seconds threshold.

S9.3 Alternative alignment scores

To mitigate some issues associated with the "audience-based" approach for estimating the relative ideological slant of news outlets, we repeated those parts of our analyses that are based on alignment scores (i.e., the analyses of ideological news diversity, the standard deviation of online news diet slant, its distributions, and the multivariate regression analyses) with exogenous scores that have been used in various previous web-tracking studies. To this end, we use a total of seven alternative scores that have been collected, made publicly available, and described in greater detail in (*63*). Since these scores are only available for American website domains, we restrict the additional analysis to the US-specific results. Specifically, we use the scores crafted from URL sharing patterns of Facebook users by (*51*). The Robertson scores rest on audience sharing behavior on Twitter (*63*). The Budak scores are rater-based scores derived through a combination of machine-learning and crowdsourcing techniques (*40*). The AllSides community scores are crowdsourced domain bias ratings aggregated by the website AllSides—an established source in US accounts of partisan bias. Specifically, AllSides provides two scores: one score that is based purely on crowdsourced ratings (AllSides community score) and another score based on a patented technology that adjusts for rater bias (AllSides controlled score). The PEW scores were adapted by Robertson (*63*) from a 2014 Pew survey on trust in news sources. These scholars also collected the MTurk scores which are based on assessments of article-level slant by coders on Amazon's Mechanical Turk.

We initially established the compatibility of these scores with the alignment scores calculated based on the present data. Looking at the panel heads in Figure S13, we see that the alignment scores derived with the present data generally showed strong to very strong correlations with the scores obtained from prior research. This was also reflected by the close correspondence of the alignment scores for the individual news websites displayed in the panels. Likewise, we see a close correspondence of both the descriptive statistical results and the regression results, which are reported in Figure S14 and Figure S16, respectively. Despite some variation in absolute levels between scores, the relative differences between both political and nonpolitical online news and between thresholds were robust.

In the same vein, the distributions in Figure S15 demonstrate a principal agreement across the alignment scores that: (A) left- and right-leaning news outlets play a greater role within US liberals and conservatives' political online news diets than their nonpolitical online news diets; (B) US liberals and US conservatives are less likely to share political online news exposure than nonpolitical online news exposure with each other; (C) US liberals' political online news diets mostly lean to the ideological left, though often only rather modestly; (D) considerable proportions of US conservatives' political online news diets lean of the ideological right and exhibit a strong right-leaning slant because they revolve around news sites like Fox News. The notable exception in the latter regard were the Mturk scores, because they attributed Fox News only

a very modest right-leaning alignment while simultaneously attributing outlets like CNN a stronger left-leaning slant than the other scores (see also Figure S13). Yet, the Mturk scores were also the ones who most strongly accentuated the right end of the ideological spectrum in the US is not confined to Fox News but news exposure yet further right also plays a role.

Figure S13: Comparison of Alignment Scores

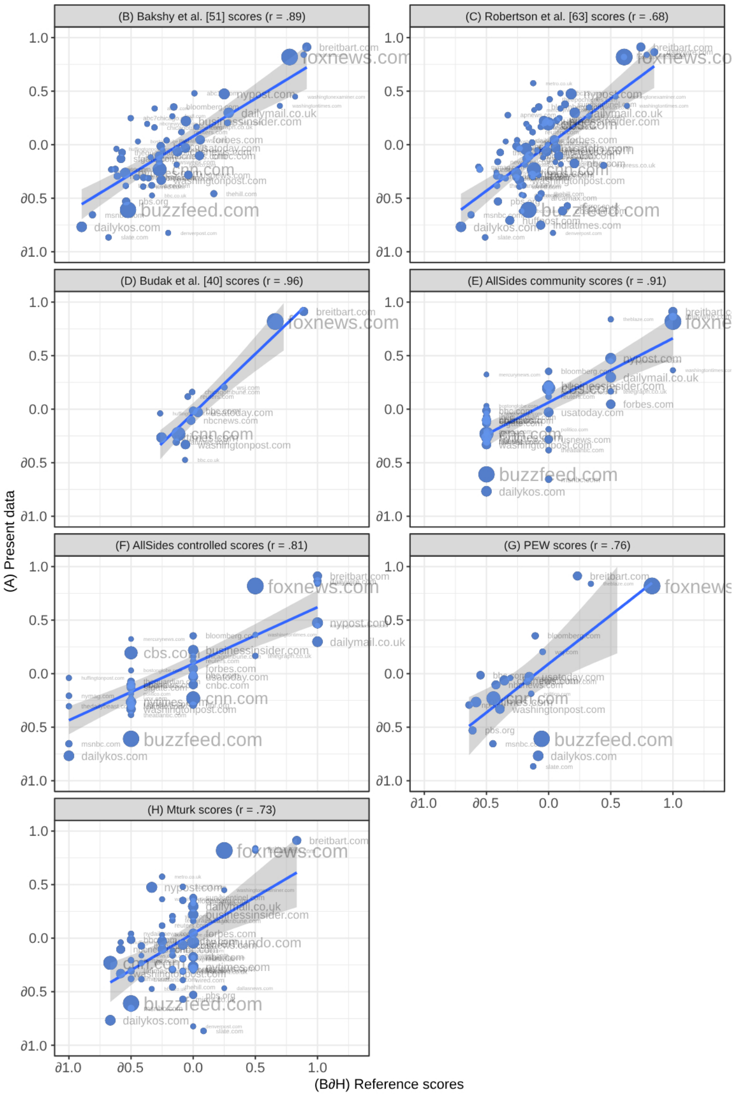

Note: Scatter plots comparing the alignment scores calculated with the present data (represented on the y-axis) and the seven reference scores obtained from previous research (represented on the x-axis and differentiated b y t he p anels). P oints r epresent i ndividual news outlets, with point size being proportional to their reach. The lines represent the associations between the respective pair of alignment scores, as formally summarized by the correlation coefficients displayed in the panel he ads. The (meta-)correlation between the alignment scores calculated with the present data on the one hand and the average of the seven alignment scores obtained from prior research on the other hand was more or less perfect, $r = .99$.

Figure S14: Ideological Diversity of US Online News Diets and Standard Deviation (SD) of Their Slant

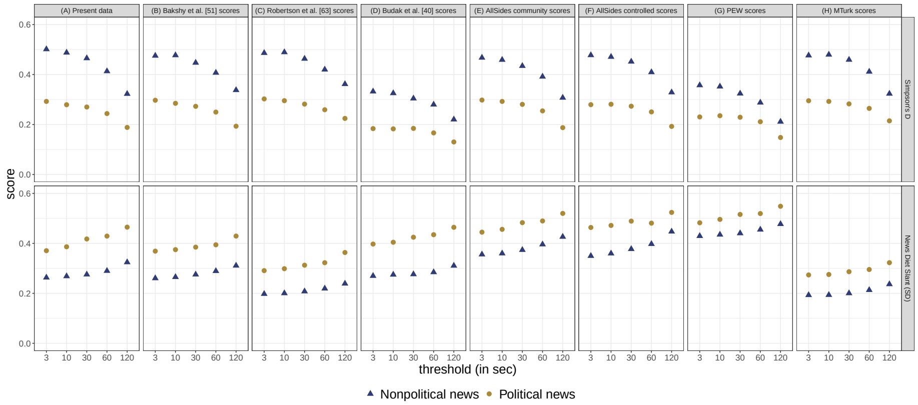

Note: The figure complements Figure 3 in the main paper by reporting the US results on ideological online news diet diversity and the standard deviation of online news diet slant obtained with seven alternative alignment scores, which are reported by Columns B to H. As a reference, we additionally included the US results generated with the present data to facilitate the comparison. These results are reported in Column A.

Figure S15: Distributions of Online News Diet Slant of US Liberals and Conservatives (Obtained with Alternative Alignment Scores)

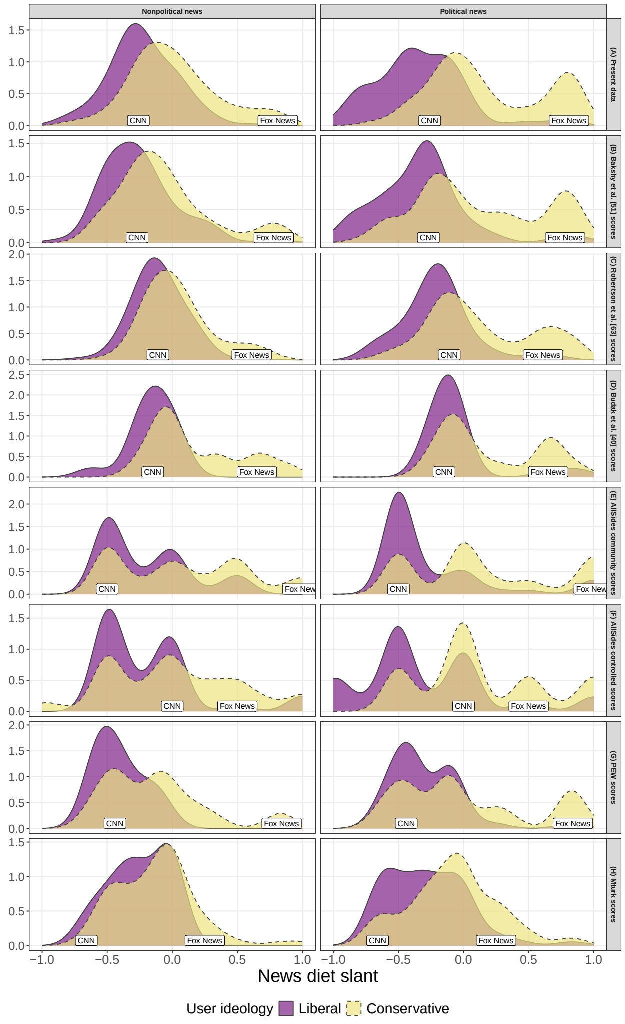

Note: The figure complements the US distributions reported in Figure 2 in the main paper with the ones obtained with the alternative alignment scores. The panels differentiate the results obtained with the different alignment scores outlined in the text.

Figure S16: Regression Results on Ideological Selectivity Online for the US

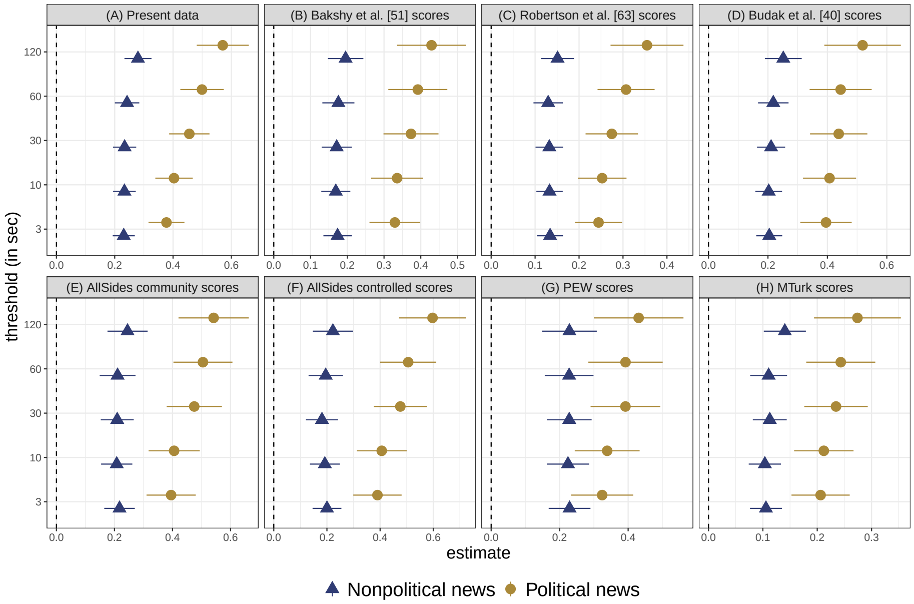

Note: Maximum-likelihood regression coefficients and 95% confidence intervals from linear multilevel models, where the focal predictor variable is ideological leaning and the dependent variables are the alignment associated with the outlet of each news visit. The panels differentiate the results obtained with the different alignment scores outlined in the text. Higher numerical values on the the x-axis represent relatively higher degrees of ideological selectivity online. Demographics (gender, age, education), political extremity, and total visits controlled but not reported.

S9.4 Multilevel analyses without controls

When it comes to the inclusion of control variables in regressions, one drawback is that the very inclusion may compromise inferences on the descriptive (mean) differences between groups like—in our case—liberals and conservatives. To address the concern, Figure S17 reports the regression estimates for the conditional differences in the slant of liberals' and conservatives' online news diets obtained when dropping the controls from the multilevel models. We see that the results on the differences between both political and nonpolitical news, temporal thresholds as well as countries, levels of political interest, and news access modes generally remained the same.

Figure S17: Conditional Differences in Liberals and Conservatives' Online News Diet Slant (Obtained from Multilevel Models without Controls)

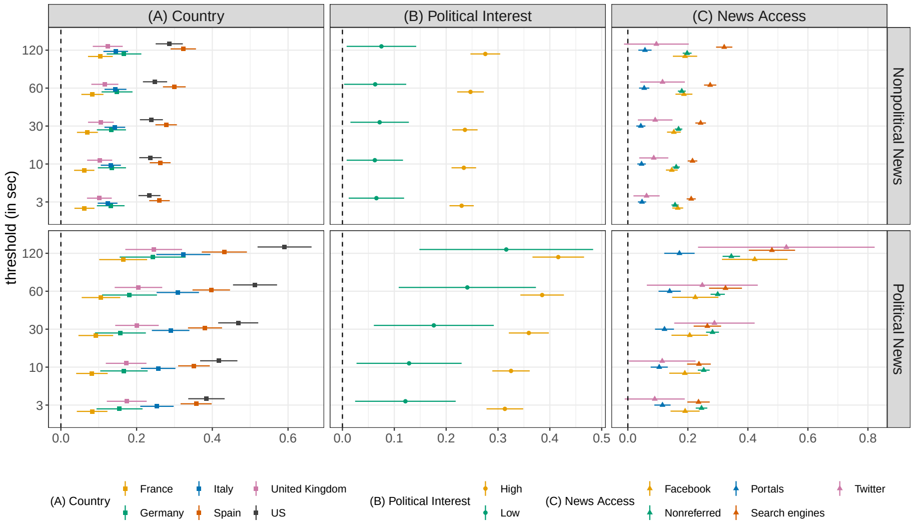

Note: The figure complements Figure 4 in the main paper by reporting the regression coefficients obtained when dropping the controls from the multilevel models.

S9.5 Multilevel analyses using raw ideology scores

The analyses reported in the main paper relied on a recoding of the raw 11-point political ideology into left-leaning, centrist, and right-leaning responses due to three main reasons. First, this approach can be regarded as a conservative standard. It safeguards that we do not only observe the differences that we observe due to cross-national differences in the preferences for extreme response categories (*76, 23, 7*). Second, the approach establishes compatibility with previous studies. Many of them have operated with a trichotomization of political ideology (*57, 17, 5*). Third, descriptive core statistics like segregation or diversity scores necessarily presuppose a categorization of ideology. As a robustness test, we still repeated the multilevel regression analyses using the raw ideology scores—with results that were perfectly consistent with our main results (Figure S18).

Figure S18: Conditional Associations Between Political Ideology and Online News Diet Slant (When Using Raw Ideology Scores)

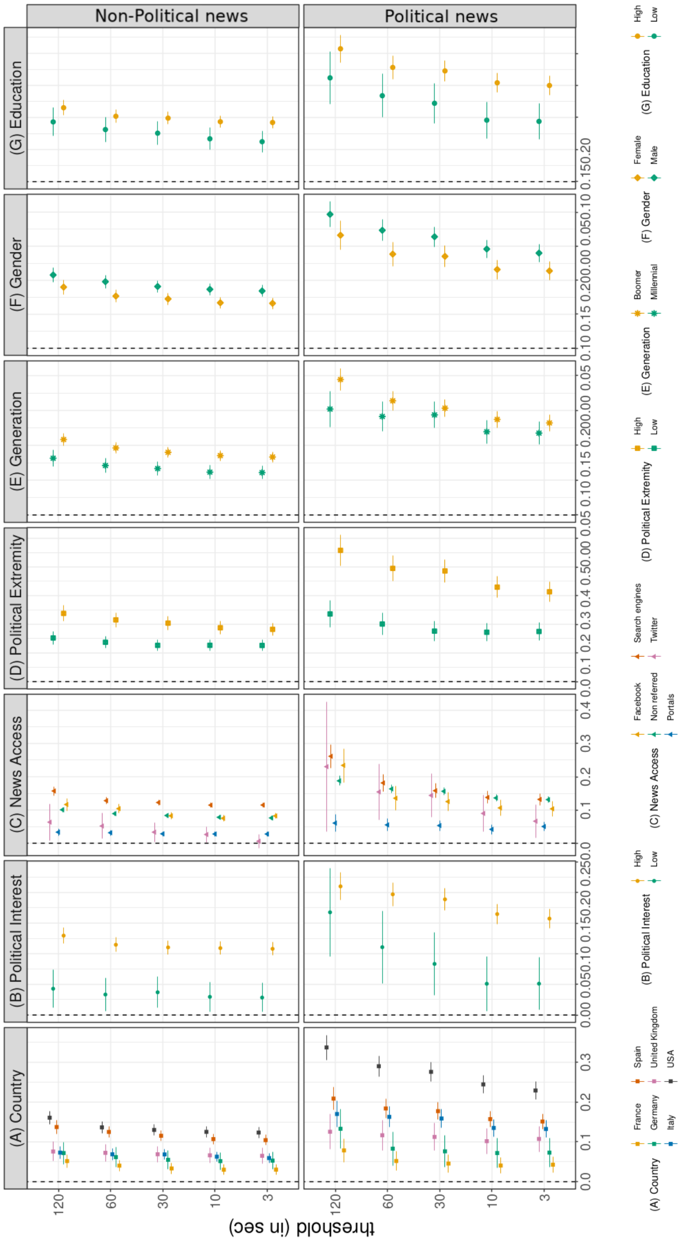

Note: Regression coefficients and 95% confidence intervals from multilevel regression models. The figure complements Figure S7 by summarizing the regression estimates obtained when using the raw 11-point political ideology scores. The generally lower absolute values of the regression coefficients compared to Figure S7 are a result of the different parameterization the raw 11-point ideology scores produce.

S9.6 Amount of online news consumption

We complemented our analysis of ideological selectivity in online news exposure with an additional test of the differences in the amount of participants' online news exposure. Given the central role of political interest in (political) news exposure (*77, 13, 6*) we aim to establish a compatibility of our study with the broader body of literature on selective exposure. We specifically accounted for political ideology by coding all left- **and** right-leaning survey responses on the ideology scale as 1 and all center responses as 0. This ensured that positive regression coefficients reflect that left- and right-leaning participants consumed more online news than participants with a centrist ideology. We denote the difference between these audience fractions with the term "ideological partisanship" in the outputs displayed in Figure S19.

Looking at the corresponding results, we see three main patterns: (A) The covariates' associations with online news exposure are in line with existing theory and empirical research. Older, male, highly educated, and politically interested participants consumed more news online. The same applied to (ideological) partisans, matching the theoretical arguments of Markus Prior (*13*). (B) The demographic and political correlates generally had stronger associations with political online news exposure than with nonpolitical online news exposure, yielding that selective exposure plays a relatively greater role with political news. (C) This specifically applied to those predictors often conceived as drivers of selective exposure in a narrower sense, i.e., political interest and ideology. They showed stronger associations with political online news exposure than with nonpolitical online news exposure, specifically for the longer temporal thresholds. (C.1) The result for political interest supported that politically interested participants were not only—as shown and discussed in detail in the main paper—overall more ideologically selective online news users. They also accounted for a greater amount of political online news exposure, both generally and even more so in terms of longer political news visits. (C.2) The role of partisanship also became stronger in longer visits of political news articles.

Figure S19: Associations of Demographic and Political Characteristics with the Amount of Online News Exposure

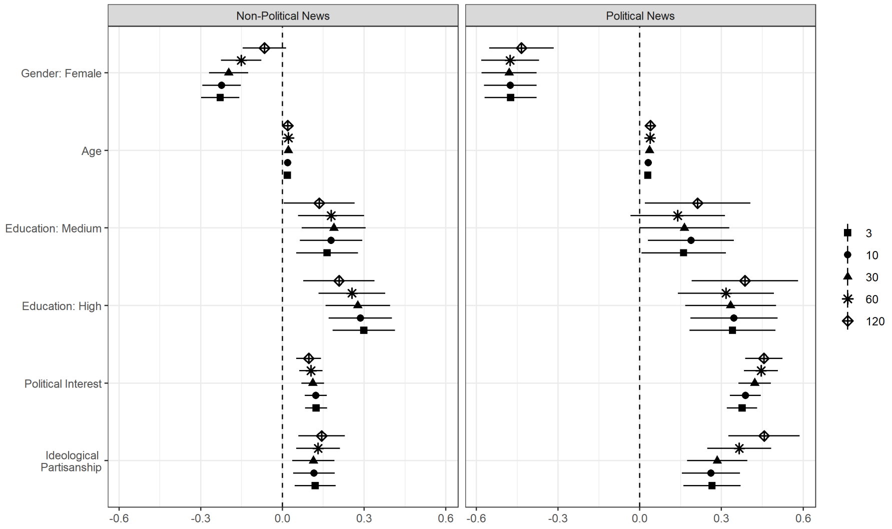

Note: Regression coefficients and 95% confidence intervals from negative binomial models, where the dependent variables are the total number of political and nonpolitical news visits over the 3-month period covered by our study, contingent on the temporal thresholds for categorizing news visits as news use episodes. The distinction between political news and nonpolitical news is represented by the panels, the thresholds are represented by the different shapes (as further explicated in the legend displayed right). "Low education" is the reference category for education, "Male" for gender. Country dummy variables and total website visits are included as controls but not reported. Our use of the term "Ideological Partisanship" and the substantive meaning of the associated regression coefficient is explained in the text.

REFERENCES AND NOTES

1. S. González-Bailón, Y. Lelkes, Do social media undermine social cohesion? A critical review. *Soc. Issues Policy Rev.* **17**, 155–180 (2023).

2. P. Lorenz-Spreen, L. Oswald, S. Lewandowsky, R. Hertwig, A systematic review of worldwide causal and correlational evidence on digital media and democracy. *Nat. Hum. Behav.* **7**, 74–101 (2022).

3. D. Muise, H. Hosseinmardi, B. Howland, M. Mobius, D. Rothschild, D. J. Watts, Quantifying partisan news diets in web and TV. *Sci. Adv.* **8**, eabn0083 (2022).

4. M. Tyler, J. Grimmer, S. Iyengar, Partisan enclaves and information bazaars: Mapping selective exposure to online news. *J. Polit.* **84**, 1057–1073 (2022).

5. T. Yang, S. Majó-Vázquez, R. K. Nielsen, S. González-Bailón, Exposure to news grows less fragmented with an increase in mobile access. *Proc. Natl. Acad. Sci. U.S.A.* **117**, 28678–28683 (2020).

6. P. van Aelst, J. Strömbäck, T. Aalberg, F. Esser, C. de Vreese, J. Matthes, D. Hopmann, S. Salgado, N. Hubé, A. Stępińska, S. Papathanassopoulos, R. Berganza, G. Legnante, C. Reinemann, T. Sheafer, J. Stanyer, Political communication in a high-choice media environment: A challenge for democracy? *Ann. Int. Commun. Assoc.* **41**, 3–27 (2017).

7. F. Mangold, M. Scharkow, Metrics of news audience polarization: Same or different? *Commun. Methods Meas.* **16**, 157–181 (2022).

8. S. Mukerjee, S. Majó-Vázquez, S. González-Bailón, Networks of audience overlap in the consumption of digital news. *J. Commun.* **68**, 26–50 (2018).

9. M. Skovsgaard, K. Andersen, Conceptualizing news avoidance: Towards a shared understanding of different causes and potential solutions. *Journal. Stud.* **21**, 459–476 (2020).

10. W. L. Bennett, S. Iyengar, A new era of minimal effects? The changing foundations of political communication. *J. Commun.* **58**, 707–731 (2008).

11. C. R. Sunstein, *Republic.com 2.0* (Princeton Univ. Press, 2009).

12. E. J. Finkel, C. A. Bail, M. Cikara, P. H. Ditto, S. Iyengar, S. Klar, L. Mason, M. C. McGrath, B. Nyhan, D. G. Rand, L. J. Skitka, J. A. Tucker, J. J. van Bavel, C. S. Wang, J. N. Druckman, Political sectarianism in America. *Science* **370**, 533–536 (2020).

13. M. Prior, *Post-Broadcast Democracy: How Media Choice Increases Inequality in Political Involvement and Polarizes Elections*, Cambridge Studies in Public Opinion and Political Psychology (Cambridge Univ. Press, Cambridge, 2007).

14. E. Pariser, *The Filter Bubble: What the Internet is Hiding From You* (Penguin Press HC, 2011).

15. S. Vaidhyanathan, *Antisocial Media: How Facebook Disconnects Us and Undermines Democracy* (Oxford Univ. Press, 2018).

16. R. Fletcher, A. Kalogeropoulos, R. K. Nielsen, More diverse, more politically varied: How social media, search engines and aggregators shape news repertoires in the United Kingdom. *New Media Soc.* **25**, 2118–2139 (2023).

17. M. Gentzkow, J. M. Shapiro, Ideological segregation online and offline. *Quarterly J. Econ.* **126**, 1799–1839 (2011).

18. M. Scharkow, F. Mangold, S. Stier, J. Breuer, How social network sites and other online intermediaries increase exposure to news. *Proc. Natl. Acad. Sci. U.S.A.* **117**, 2761–2763 (2020).

19. F. Schmidt, F. Mangold, S. Stier, R. Ulloa, Facebook as an avenue to news: A comparison and validation of approaches to identify Facebook referrals. *Polit. Commun.* 10.1080/10584609.2024.2342983, (2024).

20. E. Dubois, G. Blank, The echo chamber is overstated: The moderating effect of political interest and diverse media. *Inf. Commun. Soc.* **21**, 729–745 (2018).

21. M. Brüggemann, S. Engesser, F. Büchel, E. Humprecht, L. Castro, Hallin and Mancini revisited: Four empirical types of Western media Systems. *J. Commun.* **64**, 1037–1065 (2014).

22. D. Hallin, P. Mancini, *Comparing Media Systems: Three Models of Media and Politics*. (Cambridge Univ. Press, 2012).

23. R. Fletcher, A. Cornia, R. K. Nielsen, How polarized are online and offline news audiences? A comparative analysis of twelve countries. *Int. J. Press Polit.* **25**, 169–195 (2020).

24. E. Nechushtai, From liberal to polarized liberal? Contemporary U.S. news in Hallin and Mancini's typology of news systems. *Int. J. Press Polit.* **23**, 183–201 (2018).

25. J. T. Jost, D. S. Baldassarri, J. N. Druckman, Cognitive-motivational mechanisms of political polarization in social-communicative contexts. *Nat. Rev. Psychol.* **1**, 560–576 (2022).

26. D. A. Parry, B. I. Davidson, C. J. R. Sewall, J. T. Fisher, H. Mieczkowski, D. S. Quintana, A systematic review and meta-analysis of discrepancies between logged and self-reported digital media use. *Nat. Hum. Behav.* **5**, 1535–1547 (2021).

27. M. Scharkow, The accuracy of self-reported Internet use: A validation study using client log data. *Commun. Methods Meas.* **10**, 13–27 (2016).

28. S. Flaxman, S. Goel, J. M. Rao, Filter bubbles, echo chambers, and online news consumption. *Public Opin. Q.* **80**, 298–320 (2016).

29. A. M. Guess, (Almost) everything in moderation: New evidence on Americans' online media diets. *Am. J. Polit. Sci.* **65**, 1007–1022 (2021).

30. M. Prior, Improving media effects research through better measurement of news exposure. *J. Polit.* **71**, 893–908 (2009).

31. A. Kalogeropoulos, R. Fletcher, R. K. Nielsen, News brand attribution in distributed environments: Do people know where they get their news? *New Media Soc.* **21**, 583–601 (2019).

32. J. Möller, R. N. van de Velde, L. Merten, C. Puschmann, Explaining online news engagement based on browsing behavior: Creatures of habit? *Soc. Sci. Comput. Rev.* **38**, 616–632 (2020).

33. K. Andersen, C. H. de Vreese, E. Albæk, Measuring media diet in a high-choice environment: Testing the list-frequency technique. *Commun. Methods Meas.* **10**, 81–98 (2016).

34. R. Fletcher, R. K. Nielsen, Are news audiences increasingly fragmented? A cross-national comparative analysis of cross-platform news audience fragmentation and duplication. *J. Commun.* **67**, 476–498 (2017).

35. S. Stier, J. Breuer, P. Siegers, K. Thorson, Integrating survey data and digital trace data: Key issues in developing an emerging field. *Soc. Sci. Comput. Rev.* **38**, 503–516 (2020).

36. J. Allen, B. Howland, M. Mobius, D. Rothschild, D. Watts, Evaluating the fake news problem at the scale of the information ecosystem. *Sci. Adv.* **6**, eaay3539 (2020).

37. J. L. Nelson, J. G. Webster, The myth of partisan selective exposure: A portrait of the online political news audience. *Soc. Med. Soc.* **3**, 10.1177/20563051177293 (2017).

38. S. Iyengar, K. S. Hahn, Red media, blue media: Evidence of ideological selectivity in media use. *J. Commun.* **59**, 19–39 (2009).

39. N. J. Stroud, *Niche News: The Politics of News Choice* (Oxford Univ. Press, 2011).

40. C. Budak, S. Goel, J. M. Rao, Fair and balanced? Quantifying media bias through crowdsourced content analysis. *Public Opin. Q.* **80**, 250–271 (2016).

41. A. G. Ekström, D. C. Niehorster, E. J. Olsson, Self-imposed filter bubbles: Selective attention and exposure in online search. *Comput. Hum. Behav. Rep.* **7**, 100226 (2022).

42. A. S. Kümpel, The issue takes it all? Incidental news exposure and news engagement on facebook. *Digit. Journal.* **7**, 165–186 (2019).

43. F. Mangold, S. Stier, J. Breuer, M. Scharkow, The overstated generational gap in online news use? A consolidated infrastructural perspective. *New Media Soc.* **24**, 2207–2226 (2022).

44. M. Wieland, K. Kleinen-von Königslöw, Conceptualizing different forms of news processing following incidental news contact: A triple-path model. *Journalism* **21**, 1049–1066 (2020).

45. P. F. A. van Erkel, P. van Aelst, Why don't we learn from social media? Studying effects of and mechanisms behind social media news use on general surveillance political knowledge. *Polit. Commun.* **38**, 407–425 (2021).

46. J. K. Lee, E. Kim, Incidental exposure to news: Predictors in the social media setting and effects on information gain online. *Comput. Hum. Behav.* **75**, 1008–1015 (2017).

47. J. Seawright, J. Gerring, Case selection techniques in case study research: A menu of qualitative and quantitative options. *Polit. Res. Q.* **61**, 294–308 (2008).

48. A. S. Cardenal, C. Aguilar-Paredes, C. Galais, M. Pérez-Montoro, Digital technologies and selective exposure: How choice and filter bubbles shape news media exposure. *Int. J. Press Polit.* **24**, 465–486 (2019).

49. M. Wojcieszak, E. Menchen-Trevino, J. F. F. Goncalves, B. Weeks, Avenues to news and diverse news exposure online: Comparing direct navigation, social media, news aggregators, search queries, and article hyperlinks. *Int. J. Press Polit.* **27**, 860–886 (2022).

50. J. G. March, J. P. Olsen, *Rediscovering Institutions: The Organizational Basis of Politics* (The Free Press, 1989).

51. E. Bakshy, S. Messing, L. A. Adamic, Exposure to ideologically diverse news and opinion on Facebook. *Science* **348**, 1130–1132 (2015).

52. D. M. Cutler, E. L. Glaeser, J. L. Vigdor, The rise and decline of the American ghetto. *J. Polit. Econ.* **107**, 455–506 (1999).

53. P. Jürgens, B. Stark, Mapping exposure diversity: The divergent effects of algorithmic curation on news consumption. *J. Commun.* **72**, 322–344 (2022).

54. S. Stier, F. Mangold, M. Scharkow, J. Breuer, Post post-broadcast democracy? News exposure in the age of online intermediaries. *Am. Polit. Sci. Rev.* **116**, 768–774 (2022).

55. A. Gelman, J. Hill, *Data Analysis Using Regression and Multilevel/Hierarchical Models* (Cambridge Univ. Press, 2012).

56. Y. Krupnikov, J. B. Ryan, *The Other Divide: Polarization and Disengagement in American Politics* (Cambridge Univ. Press, 2022).

57. R. Fletcher, C. T. Robertson, R. K. Nielsen, How many people live in politically partisan online news echo chambers in different countries? *J. Quant. Descr. Digit. Media* **1**, 10.51685/jqd.2022.012 (2021).

58. M. Haim, A. Graefe, H. B. Brosius, Burst of the filter bubble? Effects of personalization on the diversity of Google news. *Digit J.* **6**, 330–343 (2017).

59. N. Newman, R. Fletcher, D. A. Levy, R. K. Nielsen, *Digital News Report 2021* (Reuters Institute for the Study of Journalism, 2021).

60. T. Johnson, P. Kulesa, Y. I. Cho, S. Shavitt, The relation between culture and response styles: Evidence from 19 countries. *J. Cross Cult. Psychol.* **36**, 264–277 (2017).

61. P. M. Blau, A macrosociological theory of social structure. *Am. J. Sociol.* **83**, 26–54 (1977).

62. E. H. Simpson, Measurement of diversity. *Nature* **163**, 688–688 (1949).

63. R. E. Robertson, S. Jiang, K. Joseph, L. Friedland, D. Lazer, C. Wilson, Auditing partisan audience bias within Google search. *Proc. ACM Hum. Comput. Inter.* **2**, 1–22 (2018).

64. European Social Survey (ESS) European Research Infrastructure Consortium, *ESS Round 8 - 2016* (Norwegian Centre for Research Data, 2017).

65. P. Barberá, How social media reduces mass political polarization. Evidence from Germany, Spain, and the US, Job Market Paper, New York University (2014), pp. 1–46.

66. N. Newman, R. Fletcher, A. Kalogeropoulos, R. K. Nielsen, *Digital News Report 2019* (Reuters Institute for the Study of Journalism, 2019).

67. H. Wickham, rvest: Easily harvest (scrape) web pages (2020).

68. L. Ou-Yang, Newspaper3k: Article scraping & curation (2013).

69. K. Benoit, K. Watanabe, H. Wang, P. Nulty, A. Obeng, S. Müller, A. Matsuo, quanteda: An R package for the quantitative analysis of textual data. *J. Open Source Softw.* **3**, 774 (2018).

70. M. Gangl, Causal inference in sociological research. *Annu. Rev. Sociol.* **36**, 21–47 (2010).

71. A. M. Guess, J. Nagler, J. Tucker, Less than you think: Prevalence and predictors of fake news dissemination on Facebook. *Sci. Adv.* **5**, eaau4586 (2019).

72. O. D. Duncan, B. Duncan, A methodological analysis of segregation indexes. *Am. Sociol. Rev.* **20**, 210–217 (1955).

73. M. J. White, Segregation and diversity measures in population distribution. *Popul. Index* **52**, 198–221 (1986).

74. D. Frankel, O. Volij, Scale invariant measures of segregation. *Documento de Debate* **8**, 1–46 (2008).

75. C. E. Shannon, A mathematical theory of communication. *Bell Syst. Tech. J.* **27**, 379–423 (1948).

76. R. Johnston, Modeling campaign dynamics on the web in the 2008 national Annenberg election study. *J. Elect. Public Opin. Parties* **18**, 401–412 (2008).

77. D. N. Hopmann, A. Wonneberger, A. Shehata, J. Höijer, Selective media exposure and increasing knowledge gaps in Swiss referendum campaigns. *Int. J. Public Opin. Res.* **28**, 73–95 (2016).
